# The Use of 3D-Printed Polymer Components for the Removal of Heavy Metals and Dyes from Water: A Systematic Literature Review

**DOI:** 10.3390/polym18091029

**Published:** 2026-04-24

**Authors:** Catarina S. P. Borges, Ana P. Piedade

**Affiliations:** University of Coimbra, (CEMMPRE), Department of Mechanical Engineering, 3030-788 Coimbra, Portugal; catarina.borges@dem.uc.pt

**Keywords:** water treatment, 3D printing, metal removal, dyes, removal of contaminants from water

## Abstract

Water is one of the most valuable resources on the planet; without it, life as we know it could not exist. Consequently, its increasing scarcity and pollution, which are mainly due to industrialization and changing consumption patterns, intensify the stress on water resources. At the same time, industrial activities contribute to water contamination with pollutants such as heavy metals, further reducing water availability. Due to their risks to human health and ecosystems, effective removal strategies are essential. Among the emerging approaches, polymer-based additive manufacturing (AM), commonly known as 3D printing (3DP), has gained attention for water treatment due to its versatility, precise control over structure and porosity, and ease of processing, while remaining at a low cost. Additionally, the polymers used have interesting adsorbent properties and allow for the incorporation of functional additives, further enhancing their performance. This review analyses the recent advances in 3D-printed polymeric materials for the removal of heavy metals and dyes, focusing on material composition, manufacturing technologies, geometry, removal mechanisms, performance, and regeneration. It was concluded that metal ions and cationic dyes are primarily removed through adsorption, due to interactions with negatively charged surfaces that are often enhanced by high-affinity additives. Anionic dyes are generally less effectively removed by adsorption and often rely on degradation mechanisms. However, adsorption of anionic dyes can occur, for instance when the adsorbent surface is modified to introduce positively charged functional groups. The ability of 3DP to create hierarchical porous structures combining micro-, meso-, and macropores improves fluid flow and contact area, thereby enhancing the removal efficiency.

## 1. Introduction

Fresh water is one of the world’s most vital resources, and is essential for human and ecosystem survival, as well as for agricultural and industrial activities. However, its availability has declined due to both increasing demands, driven by population growth and the intensification of industrial activities, and decreasing supply, resulting from the growing pollution of water systems [1].

Water pollution has been identified by the World Economic Forum as one of the major risks for the coming years [2], as it places additional pressure on the already limited freshwater resources. This degradation is primarily driven by industrial activities and wastewater discharges into the grid, which lead to the release of harmful pollutants into water bodies, such as pesticides, surfactants, pharmaceutical residues, and industrial waste, including both organic and inorganic compounds such as dyes and heavy metals.

Heavy metals are among the most dangerous water pollutants, due to their non-biodegradability and tendency to bioaccumulate [3]. Although there is no universally accepted definition, they are generally described as naturally occurring elements with high density (exceeding 5 g·cm^−3^) and atomic mass [4]. Common examples include arsenic (As), lead (Pb), chromium (Cr), mercury (Hg), cobalt (Co), cadmium (Cd), nickel (Ni), vanadium (V), manganese (Mn), copper (Cu), and zinc (Zn). Arsenic, lead, mercury, and cadmium are linked to carcinogenic effects; neurological, respiratory, and gastrointestinal damage; and impaired physiological functions, and can ultimately be fatal [5,6,7,8]. Other metals, such as copper, zinc, chromium, nickel, cobalt, and vanadium, are associated with genetic, cardiovascular, and respiratory damage, as well as anemia and cognitive malfunctions [6,9,10,11,12]. Dyes can exhibit carcinogenic, mutagenic, and genotoxic effects, potentially causing DNA damage, as well as skin irritation, allergic reactions, respiratory issues, and liver and kidney damage [13,14]. Aside from their direct impact on human health, both heavy metals and dyes exert significant ecotoxic effects, disrupting aquatic ecosystems, harming flora and fauna, and bioaccumulating along food chains. Therefore, their removal from water is essential.

Conventional water treatment methods are often insufficient, as they often cannot completely remove the target contaminants on a large scale, while also facing challenges such as high energy consumption, the use of non-degradable chemical products, and the generation of secondary waste [15], which emphasize the importance of developing alternative decontamination or remediation strategies.

Polymers have been proposed as a solution for water treatment procedures due to their ease of processing, high specific mechanical properties, and low cost [16,17]. Some polymers, such as alginate or cellulose, already offer adsorption sites for water treatment, and they are frequently combined with reinforcements, including other polymers as well as metallic or ceramic materials, which enable the tuning and enhancement of mechanical properties and of physical and chemical interactions with contaminants for water treatment applications. Therefore, in these applications, polymers can act as the active adsorbent, as a support for active adsorbents, such as powders, nanoparticles or fibers, or fulfill both roles simultaneously.

To process these polymers, AM technology has attracted attention, as it enables the production of complex structures while offering advantages in terms of automation, scalability, cost-effectiveness, and reduced material waste [18]. However, 3DP also presents general limitations, including the frequent need for post-processing, time-consuming production at large scales, limited resolution, and, for some technologies, a restricted range of available materials [19,20]. However, some of the drawbacks of 3DP can be advantages in the context of contaminant removal, particularly because of the increased defects (porosity) associated with the process.

In this paper, the systematic literature review focused on 3D-printed polymeric materials for removing heavy metal and dye contaminants from water is presented. First, the methodological approach is discussed, followed by the AM technologies used, contaminant removal methods, material design and geometries, and finally future perspectives and conclusions.

## 2. Methodology

This systematic literature review analyses 3D-printed polymer-based structures for the removal of heavy metals and dye contaminants from water (including wastewater and other aqueous systems). The research question was established as being “how do 3D-printed polymer-based structures remove heavy metals and dyes from water, and how do material chemistry, printed architecture, and operating conditions influence their performance and limitations?”. The population (P) was defined as water contaminated with heavy metals and/or dyes, the intervention (I) as 3D-printed polymeric structures, the comparison (C) was not applicable, and the outcomes (O) included removal efficiency, adsorption isotherms, kinetics, regeneration, or other performance indicators. The PRISMA 2020 guidelines were followed (Table S1) to identify the relevant papers. The initial search was conducted on the 2nd of February 2026 and updated until the 26th of February 2026. The final dataset corresponds to the complete search period. Records were identified from two scientific databases—Web of Science and Scopus—using the queries presented in Table S2. A note should be made regarding oil contaminants, which were identified during the initial search and subjected to the same inclusion and exclusion criteria prior to full-text screening. These studies were subsequently excluded during full-text screening to improve the coherence and conciseness of the review, as the mechanisms governing oil removal differ from those associated with heavy metals and dyes and are evaluated using different performance metrics. Including them would therefore result in a loss of focus and reduce the potential for meaningful comparisons across studies.

Eligibility criteria were defined to identify studies that were relevant to polymer-based AM applications for water treatment. Studies were included if they: (i) were experimental research articles written in English; (ii) employed AM (3DP) to fabricate structures involved in the treatment process; (iii) focused on polymer-based materials, including composite, functionalized, or coated systems; (iv) addressed the treatment of water, wastewater, or other aqueous solutions; (v) targeted heavy metal or dye contamination; and (vi) reported quantitative contaminant removal or degradation performance. Studies were excluded if they did not meet these eligibility criteria, including non-experimental publications (e.g., reviews), studies not involving AM, studies focusing only on non-polymeric materials, or studies lacking quantitative performance data. Duplicate records and records without accessible full text were removed during the screening process. The number of documents considered and eliminated at each stage is outlined in Figure S1.

**Figure 1:**
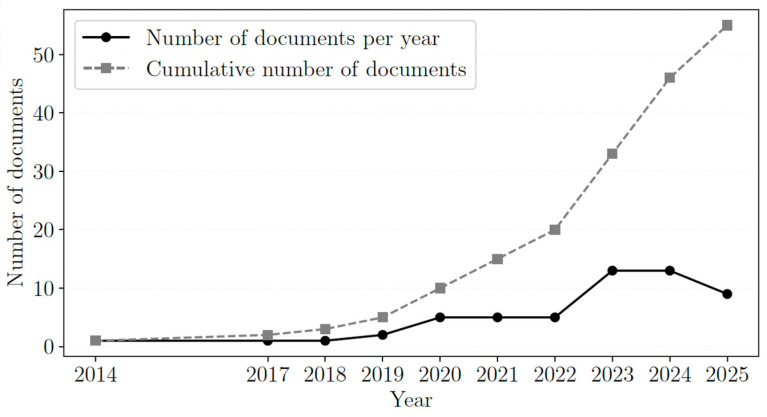
Distribution of the reviewed studies by publication year.

At the end of the screening process, 59 documents were included, of which 28 addressed heavy metal removal and 28 addressed dye removal, and three documents covered both topics. The distribution of the papers by publication year is shown in Figure 1. The results indicate that this is a growing research topic. Although a slight decrease in the number of new publications is observed in 2025, additional data from subsequent years are required to determine whether this represents a change in the overall trend. Publications from 2026 were not included in the plot, as only the first two months of the year were considered.

A quality assessment of the included studies was conducted based on the following criteria: (i) material description (30%; 0—none, 1—material family reported, 2—specific material identified), (ii) 3DP technology used (30%; 0—none, 1—technology family reported, 2—specific technology identified), (iii) performance metrics (20%; 0—other or not specified, 1—removal capacity or removal efficiency reported, 2—both reported), (iv) kinetic and isotherm analysis (10%; 0—none, 1—one of them reported, 2—both reported), and (v) regeneration performance (10%; 0—none, 1—number of regeneration cycles reported, 2—number of cycles and capacity retention reported). A summary of the included studies is presented in Tables S3 and S4 for heavy metals and dyes, respectively, including the quality score.

## 3. Additive Manufacturing Technologies Used for Water Treatment Applications

**Figure 2:**
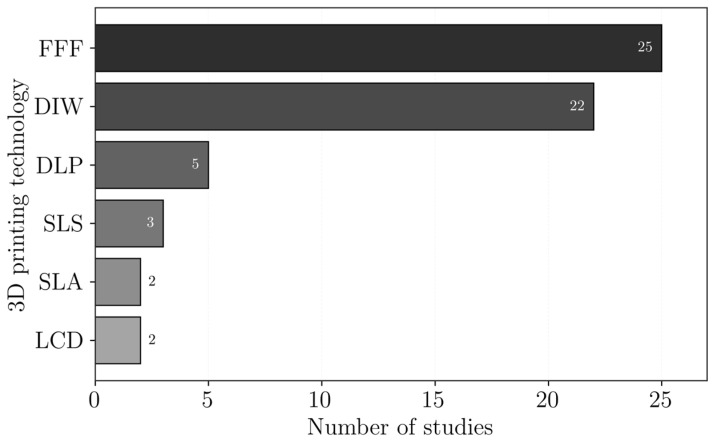
Distribution of 3DP technologies used in the reviewed studies: fused filament fabrication (FFF), direct ink writing (DIW), digital light processing (DLP), selective laser sintering (SLS), stereolithography (SLA), and liquid crystal display (LCD).

The 3DP technologies reported in the reviewed studies are presented in Figure 2. It should be noted that not all authors clearly state the 3DP technology used, and, in some cases, it had to be inferred from the description of the process. The most common processes are material extrusion, followed by vat photopolymerization, and, finally, powder bead fusion, specifically selective laser sintering technology.

**Figure 3:**
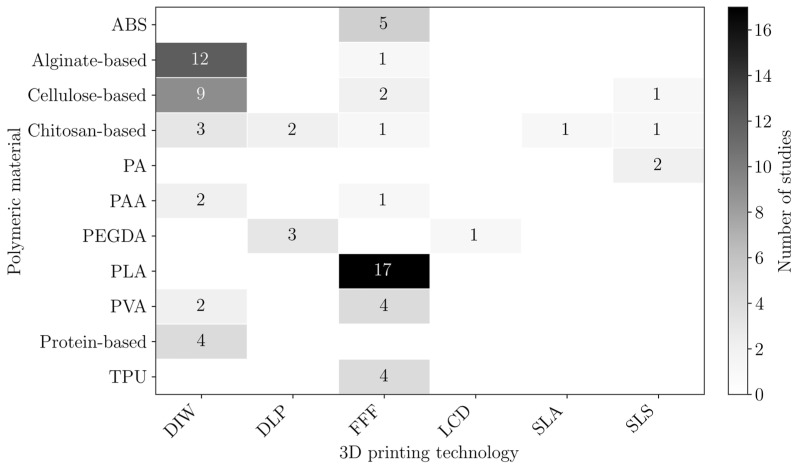
Distribution of polymers across the different 3DP technologies reported in the reviewed studies (including poly(acrylonitrile–butadiene–styrene) (ABS), polyamide (PA), poly(acrylic acid) (PAA), poly(ethylene glycol diacrylate) (PEGDA), poly(lactic acid) (PLA), poly(vinyl alcohol) (PVA) and polyurethane (TPU)).

The combinations of the used polymers and the 3DP technologies employed are presented in Figure 3. Polymers that were only reported once were excluded from this plot to improve readability and facilitate the identification of trends. Additionally, polymers used as a matrix or reinforcements were not distinguished in this plot.

### 3.1. Extrusion-Based 3D Printing

Extrusion-based AM technologies produce components by the controlled layer-by-layer deposition of material via a nozzle or syringe. These technologies enable faster fabrication cycles compared to other AM technologies, which also shows high versatility. Additionally, extrusion-based 3DP devices are widely available, making them the most used process family for the fabrication of 3D-printed wastewater treatment components. The extrusion-based technologies reported for this application were fused filament fabrication (FFF) and direct ink writing (DIW).

In FFF, objects are produced by pushing a polymeric-based filament through a feeding system, heating and melting it, and finally forcing it through a nozzle [21]. Therefore, this process requires the filaments to be manufactured from thermoplastic materials, which can be melted and reshaped [22,23]. FFF is widely available, compared to most AM technologies, and is compatible with a significant range of materials and reinforcements [24]. However, it may present limitations in terms of long-term stability for water treatment [25]. In the reviewed studies, this process was most often used with PLA, followed by ABS, TPU and PVA (Figure 3).

DIW, or robocasting, is a process in which a viscous ink, or paste containing fine solid particles, is deposited layer-by-layer under applied pressure. This technology has the advantage of being compatible with a broad range of materials, including polymers, hydrogels, ceramics, metals, and composites, provided that they have the suitable rheological properties [26]. However, the quality of the final object is highly dependent on the properties of the inks, requiring careful formulation [27]. Additionally, the structural stability of the printed object may depend on post-curing or other post-processing steps [28,29]. In the reviewed studies, DIW was mainly used to print inks containing alginate and cellulose (Figure 3), as it enables their processing, also allowing for the incorporation of functional fillers such as metal–organic frameworks (MOF) or graphene oxide (GO) to enhance the adsorption performance.

### 3.2. Vat Photopolymerization

Vat photopolymerization is a process in which a liquid resin is selectively cured through exposure to light, typically ultraviolet (UV), resulting in the layer-by-layer solidification of the material. This technique enables the fabrication of structures with high resolution and surface quality. However, it limits the range of usable materials, as it requires photocurable resins, and it is a relatively slow process. Post-processing steps, such as cleaning and post-curing, are often required [30,31]. The vat photopolymerization processes reported for the fabrication of components for contaminant removal comprehend stereolithography (SLA), digital light processing (DLP) and liquid crystal display (LCD)-based vat photopolymerization.

SLA is a vat photopolymerization technique in which a liquid resin is selectively cured by a focused UV laser. The laser scans the resin surface according to the cross-sectional geometry of each layer [32]. SLA, in the reviewed documents, was used with chitosan, crosslinked acrylic networks and thiolyne polymers. In DLP, instead of a laser, a digital micromirror device projects a light pattern that cures an entire layer of photopolymer resin at once, enabling faster printing speeds [33]. DLP was reported for systems containing PEGDA, chitosan and acrylic-based polymers. In LCD-based vat photopolymerization, a light source is positioned beneath the resin vat, while an LCD screen acts as a mask that selectively blocks or transmits light to cure the desired regions of each layer [34]. LCD-based vat photopolymerization was used, in the reviewed studies, with a system containing a non-identified photocurable resin and polyvinyl phosphonic acid (PVPA).

### 3.3. Selective Laser Sintering (SLS)

SLS is a powder bed fusion process in which polymer powder particles are fused using a high-energy laser to form solid components. This process can produce mechanically robust parts with controlled porosity and does not require support structures, as the surrounding not-sintered powder provides support during printing. However, the range of materials that can be used is limited, as they must be available in powder form and possess suitable thermal properties for processing [35]. In the selected studies, SLS is used with systems containing polymers, such as cellulose, chitosan, polystyrene (PS) and PA, with and without MOF additives.

## 4. Mechanisms of Contaminant Removal

**Figure 4:**
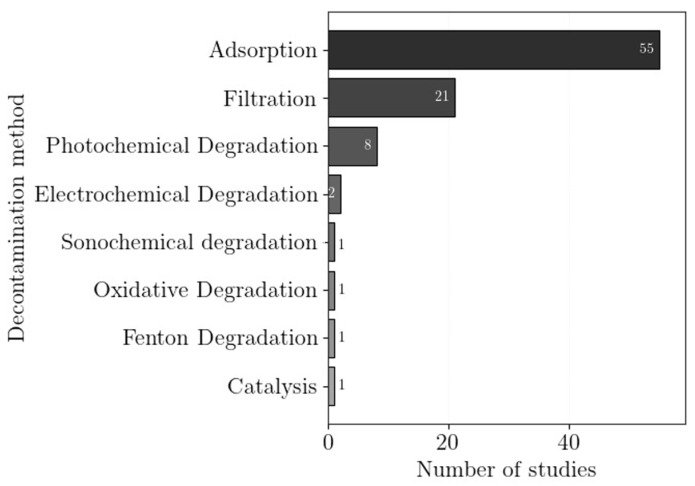
Most common decontamination methods reported in the reviewed studies.

The decontamination methods reported in the reviewed studies are shown in Figure 4. Adsorption was the most employed method, due to its high removal efficiency, simplicity and low cost (Section 4.2). Several studies reported photochemical degradation (Section 4.3.1), as well as other degradation processes, such as Fenton (Section 4.3.2), electrochemical (Section 4.3.3), sonochemical (Section 4.3.4), and other oxidative methods (Section 4.3.5), both working independently and paired with adsorption. Filtration and electrostatic exclusion (Section 4.4) were also used as removal strategies.

### 4.1. Contaminants Removed

**Figure 5:**
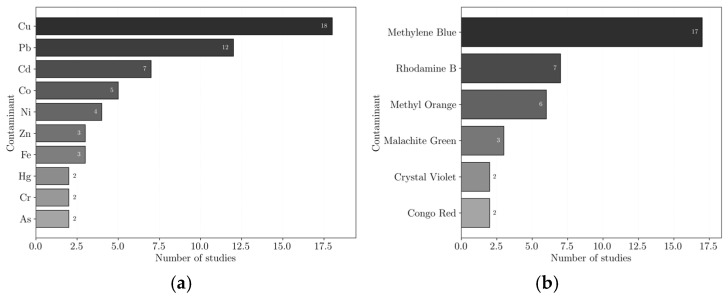
Contaminants reported for removal in the reviewed studies: (**a**) heavy metals and (**b**) dyes.

To understand the removal mechanisms used, first, it is important to know the contaminants reported for removal in the reviewed studies presented in Figure 5a,b for heavy metals and dyes, respectively. Contaminants which only appeared in one study were neglected to improve readability.

Regarding heavy metals, the most common targets were copper (Cu) and lead (Pb), followed by cadmium (Cd), cobalt (Co), nickel (Ni), zinc (Zn), iron (Fe), mercury (Hg), chromium (Cr) and arsenic (As). When in water, these species often appear as cations, in the form of copper (Cu^2+^) and lead (Pb^2+^), cadmium (Cd^2+^), cobalt (Co^2+^), nickel (Ni^2+^), zinc (Zn^2+^), iron (Fe^2+^/Fe^3+^), mercury (Hg^2+^), chromium (Cr^6+^/Cr^3+^), and arsenic (As^3+^/As^5+^).

Regarding dyes, the most addressed contaminants included Methylene Blue, Rhodamine B, Methyl Orange, Malachite Green, Crystal Violet, and Congo Red. Dyes are commonly classified as cationic or anionic based on the electrical charge they possess in aqueous solution, which can be positive or negative, respectively [36,37]. Cationic dyes, such as Methylene Blue, Rhodamine B, Malachite Green, and Crystal Violet, tend to interact with negatively charged surfaces, which is why they are often easily removed by adsorption. Anionic dyes, such as Methyl Orange and Congo Red, can be more difficult to remove by adsorption, unless the adsorbent structure is modified and positively charged; therefore, additional degradation methods were often required.

### 4.2. Adsorption

The most common mechanism for contaminant removal from water is adsorption, due to its low cost, high removal efficiency, ease of use, versatility, eco-friendliness, compatibility with continuous-flow systems and regeneration possibility [38,39]. Adsorption may be caused by physical or chemical interactions [40].

#### 4.2.1. Physical Phenomena

Physical mechanisms include electrostatic attraction, hydrogen bonding, van der Waals forces, hydrophobic interactions and π–π interactions.

Electrostatic attraction occurs between oppositely charged adsorbent surfaces and functional groups or ionic contaminants [41]. In the case of the polymer surfaces used in water treatment, they are typically negatively charged, due to the deprotonation of functional groups such as carboxyl (–COOH), sulphonic acid (–SO_3_H) and phosphoric acid (–H_3_PO_4_), forming the corresponding anionic species (–COO^−^, –SO_3_^−^, and –PO_4_^3−^). For instance, natural polymers, such as alginate or cellulose [42,43,44], can exhibit a negative charge in water at a neutral pH, with the magnitude of the negative charge increasing as the pH increases, attracting positive metal ions and cationic dyes. These materials can also be chemically modified or surface-treated to increase their negative surface charge, and particles that are rich in these functional groups may be introduced to further improve adsorption [45,46,47]. The inverse is also true, between positively charged surfaces and negative ions.

Hydrogen bonding occurs when a hydrogen atom, bonded to a more electronegative atom or group, such as hydroxyl (–OH), amine (–NH_2_), sulfonate (–SO_3_^−^), or carboxyl (–COOH) in the adsorbent, interacts with another electronegative atom possessing a lone pair of electrons [48]. Therefore, it is more common for dye contamination [49,50,51]. In the case of metal contamination, it can have an indirect role, interacting with water molecules surrounding the metal ion and stabilizing it near the surface to improve adsorption. Another type of physical interaction contributing to adsorption is van der Waals forces, which arise from temporary shifts in electron density within atoms or molecules, and create temporary dipoles, which induce dipoles in neighboring species, contributing to their attraction [52]. This mechanism is also more relevant for dye contamination, stabilizing the contaminant on the surface of the adsorbent.

Hydrophobic interactions can also contribute to dye removal, particularly for dyes containing aromatic rings or non-polar domains [53], since hydrophobic regions of the dye molecule are associated with hydrophobic regions on the adsorbent surface, minimizing contact with water and making adsorption energetically favorable, promoting the attachment of the dye to the surface, rather than remaining dissolved in water.

π–π interactions are interactions between aromatic rings in the adsorbent and in the contaminant [54]. When the adsorbent and adsorbate approach each other, for instance due to electrostatic interactions, there is an interaction between their π–electron clouds, which results in the stabilization of the dye molecule on the surface, contributing to adsorption [53]. Therefore, this is also an adsorption phenomenon that can only play a role with dye contaminants. However, cation–π interactions may also contribute to adsorption when positively charged species, such as metal ions, interact with electron-rich aromatic rings on the adsorbent surface [55].

Porous adsorbent structures provide numerous adsorption sites, enabling the contaminant to accumulate not only on the surface of the adsorbent but also in its pore network. Therefore, many studies highlight the relevance of having a hierarchical pore network, with macropores which can be created through 3DP, enabling the fluid flow and transport of contaminants to encounter the material, and the meso- and micropores in the material itself provide the adsorption sites [56,57,58].

#### 4.2.2. Chemical Phenomena

Chemical adsorption involves processes such as ion exchange, coordination, and chelation with functional groups.

Ion exchange occurs when ions in the aqueous solution replace the mobile ions that are bound to the adsorbent’s charged functional groups [59,60]. In this case, when the polymer is placed in contact with contaminated water, the previously mentioned polymer’s charged functional groups (e.g., –COO^−^, –SO_3_^−^, and –PO_4_^3−^) exchange their initial counter-ions (e.g., H^+^, Na^+^, Ca^2+^) with metal ions that are present in the solution, such as Pb^2+^, Cu^2+^, and Zn^2+^) [61,62,63,64].

Coordination takes place when functional groups on the adsorbent, such as hydroxyl (–OH), carboxyl (–COOH) or amine (–NH_2_), donate lone pairs of electrons to the metal ion or to electron-deficient sites on dye molecules, forming a covalent bond. When bonds are established with more than one donor atom from the same molecule, a chelation complex is formed [65,66]. Coordination is crucial in metal ion adsorption, as they possess empty orbitals and a positive charge, behaving like Lewis’s acids [42,67,68].

These mechanisms are not independent and, in most cases, act in synergy. Therefore, in summary, porous structures containing functional groups such as hydroxyl (–OH), carboxyl (–COOH), sulphate (–SO_3_H), phosphate (–PO_4_H), or amine (–NH_2_) are suitable for the removal of metals and dyes, mostly of metal ions and cationic dyes, from water through adsorption. Anionic dyes are typically removed with lower efficiency; however, adsorption can still take place, for instance when the adsorbent surface is altered to incorporate positively charged functional groups. This process is undoubtably the most common for contaminant removal and has been used alone, with good removal efficiencies, or paired with other removal processes, such as degradation processes and filtration.

The analysis of adsorption kinetics and isotherm models plays a key role in evaluating the performance of adsorbent materials in water treatment applications, providing insights into their adsorption mechanisms, capacity, and efficiency. Therefore, it is crucial to understand what these models describe.

#### 4.2.3. Equilibrium Adsorption Isotherms

Adsorption isotherms describe the equilibrium relationship between adsorbates and adsorbent materials at a constant temperature [69]. To establish these isotherms, adsorption experiments are performed by exposing a fixed mass of adsorbent to solutions with different initial concentrations of the adsorbate until equilibrium is reached. Then, the isotherm is obtained by plotting the equilibrium adsorption capacity against the equilibrium concentration and fitting the isotherm model to the experimental data. In Tables S3 and S4, the isotherms reported for each study considered, when reported, are presented for heavy metals and dyes, respectively. The most common adsorption models were Langmuir and Freundlich isotherms. On the one hand, the Langmuir model assumes monolayer adsorption on a homogeneous surface [70]. On the other hand, the Freundlich model can be applied to multilayer adsorption on heterogeneous surfaces [71]. Therefore, these models enable the understanding of adsorption mechanisms and allow for the estimation of the maximum adsorption capacity of the adsorbent.

#### 4.2.4. Adsorption Kinetics

Adsorption kinetics describe the rate of the adsorption process, indicating how adsorption progresses over time and the time required to reach equilibrium [72]. To determine the most appropriate adsorption kinetic model, a fixed mass of adsorbent is exposed to a solution containing the adsorbate for different contact times. The adsorption capacity is plotted against the contact time, and kinetic models are then fitted to the experimental data. The most common kinetics models are Pseudo-first order and Pseudo-second order. The kinetics models that best fit the experimental data in the considered studies, when that analysis was done, are presented in Tables S3 and S4 for heavy metals and dyes, respectively. Pseudo-first order assumes that the adsorption rate is proportional to the number of unoccupied sites and is often associated with physisorption or early stages of adsorption [73]. Pseudo-second order, the kinetic model reported by most of the studies considered, assumes that the adsorption rate is proportional to the square of the number of unoccupied sites, and often indicates chemisorption processes [74].

#### 4.2.5. Effect of pH and Concentration of the Adsorbate

The adsorption efficiency of the ion removal depends strongly on the conditions of the aqueous environment, with the pH and concentration of the adsorbate being two of the most influential parameters.

Several studies have investigated the effect of the pH, and typically report that at a low pH, below pKa, excess H^+^ in the water competes with cations for adsorption sites, leading to reduced adsorption capacity. Additionally, the protonation of functional groups such as carboxyl (–COOH), hydroxyl (–OH), and amine (NH_2_) reduces the number of available active sites, making the surface less favorable for binding cations and weakening the electrostatic interaction between the adsorbent and the cations, or even causing repulsion. As the pH increases, these functional groups become deprotonated, which increases the attraction and coordination with cations, with an increasing number of available active sites for adsorption. Additionally, there are less ions in the solution competing for those active sites. At very high pH, the apparent removal efficiencies may increase due to the formation of cation hydroxide species (M(OH)_x_) that precipitate from the solution, which can lead to an overestimation of the actual adsorption capacity of the material [45,62,75]. Therefore, one way to reverse adsorption is to lower the pH, which protonates surface functional groups and weakens their interaction with cations, promoting desorption. This is why most regeneration procedures reported included a hydrochloric acid (HCl) treatment [42,55,76].

Additionally, as the concentration of the adsorbate in the solution increases, adsorption tends to increase due to the higher probability of collisions between the adsorbate molecules and the active sites on the adsorbent, which facilitates overcoming the mass transfer resistance of the adsorbate toward the adsorbent [46,47].

### 4.3. Degradation Processes

The degradation processes reported comprehend photochemical degradation, Fenton degradation, electrochemical degradation and sonochemical degradation.

#### 4.3.1. Photochemical Degradation

Photochemical degradation is the most common degradation process used to remove dye contaminants from water. This process is triggered by a light source. In this case, the polymeric material needs to incorporate a photocatalyst that is capable of absorbing light and generating reactive charge carriers. The catalyst material absorbs light, forming electron–hole pairs. Then, the generated electrons reduce the oxygen available on the surface, generating superoxide radicals (O_2_^•−^). Afterwards, the dye can react with water molecules or hydroxyl ions, forming hydroxyl radicals (•OH). Finally, the highly reactive radicals •OH and O_2_^•–^ govern the photodegradation process, degrading the dye into smaller products, such as CO_2_ and H_2_O [77,78]. This process was used to complement the adsorption removal of cationic dyes, as well as removing the anionic dyes which had a much lower adsorption efficiency [79,80,81].

#### 4.3.2. Fenton Degradation

The process of Fenton degradation is like photochemical degradation, differing in how the reactive radicals are generated. While in photochemical degradation, the ions are generated by the light activation of a photocatalyst, in Fenton degradation, the process is based on the reaction between ferrous ions (Fe^2+^) and hydrogen peroxide (H_2_O_2_), which produces the hydroxyl radicals (•OH). Then, the process is similar: the radicals attack the dye molecules, breaking them into smaller products. This process has been used in a particular case for the removal of Methylene Blue, with excellent efficiency [82] at a low pH.

#### 4.3.3. Electrochemical Degradation

For electrochemical degradation, activation comes from an applied electrical current, generating oxidation reactions at the anode surface. Then, there are multiple possible paths for degradation. In direct electrochemical oxidation, the process occurs directly at the anode surface through electron transfer between the electrode and the adsorbed dye molecule. There is also the possibility of water oxidation at the anode, which generates hydroxyl radicals (•OH) and, consequently, dye degradation in the solution. Finally, other species, such as chloride ions in wastewater, can be oxidized at the anode, producing soluble chlorine. Chlorine reacts with the water, breaking down into hypochlorous acid (HOCl) and hypoclorite (ClO^−^) [83,84,85]. In this case, these are the primary oxidizing agents responsible for breaking down dyes.

Electrochemical degradation was reported in two consecutive studies [86,87] for the degradation of Methyl Orange and Methylene Blue.

#### 4.3.4. Sonochemical Degradation

Sonochemical degradation is based on ultrasound irradiation which, as it propagates through the liquid, generates acoustic cavitation. Acoustic cavitation comprehends the creation, growth and collapse of microbubbles. The violent collapse of these bubbles generates extremely high temperatures and pressures. Under these conditions, water molecules can dissociate, forming reactive radicals such as •OH and H•. Then, a similar oxidation and degradation process of the dyes takes place. This process was used to degrade Rhodamine B [88].

#### 4.3.5. Other Oxidative Degradation Processes

Another oxidative degradation process is used in a particular work to degrade anionic Acid Orange 7 [89]. This process relied on a sulfonated polypropylene (PP) structure in contaminated water, to which potassium persulfate was added. The presence of the sulphonated polymer promotes the cleavage of the O–O bond, generating highly reactive sulphate radicals (SO_4_^•−^), and possibly other secondary species. Then, the sulphate radicals degrade the dye into smaller products.

These processes, although highly effective, particularly in increasing the removal of anionic species, require external activation, and can lead to secondary pollution, limiting their large-scale application in water treatment.

### 4.4. Filtration and Electrostatic Exclusion

Filtration, in its simplest form, is based on size exclusion, where a porous material with sufficiently small pores retains contaminants larger than the pore size while allowing water and smaller species to pass through [90]. Filtration is often combined with chemical precipitation, as precipitation converts dissolved contaminants into insoluble particles that can subsequently be retained by the filter pores.

Electrostatic exclusion can further increase the selectivity of filters. When the surface of the filter has electrical charges, it can repel contaminants with the same charge, blocking them for passing through the pores [90,91].

Filtration was used paired with adsorption and electrostatic exclusion to remove dyes and metal ions from contaminated water [54,92,93].

## 5. Material Design of 3D-Printed Polymer-Based Treatment Systems

### 5.1. Heavy Metal Removal

**Figure 6:**
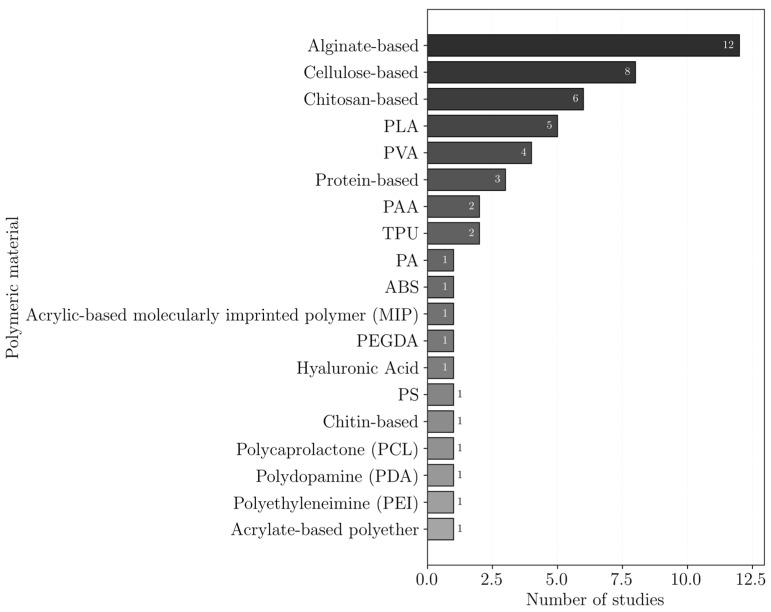
Polymers reported in the heavy metal contamination reviewed studies.

The polymers reported for heavy metal removal are shown in Figure 6. In this plot, polymer occurrences are not distinguished according to their role as matrices or additives, and all reported polymers in the studies are considered together. The results indicate a clear preference for more sustainable and biodegradable polymers, such as alginate, cellulose, chitosan, PLA, PVA, and protein-based polymers.

**Figure 7:**
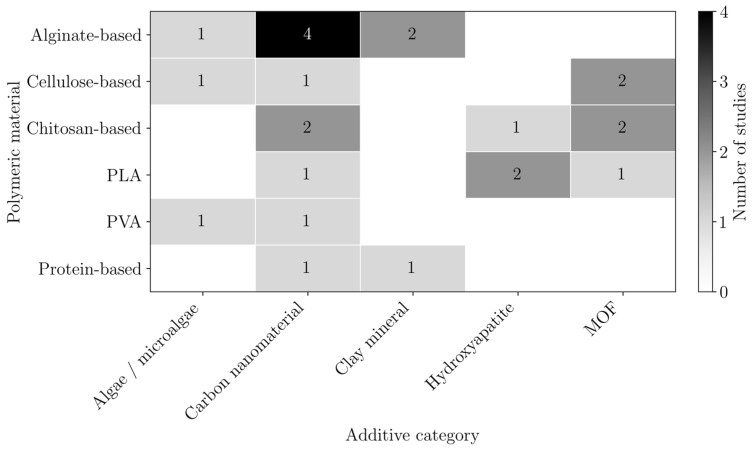
Polymers and additives reported in the heavy metal contamination reviewed studies.

These polymers may act as active adsorbents, support materials, or both. In many cases, they are combined with high-affinity additives to enhance adsorption performance, as shown in Figure 7. The most used additives were carbon nanomaterials, particularly GO, with or without modification, and MOFs, both of which have large surface areas.

GO is a highly effective adsorbent with abundant oxygen-containing functional groups [47,58]. MOFs are highly ordered porous solids constructed from metal nodes (ions or clusters) connected by organic linkers, forming a highly porous network. These materials typically exhibit tunable pore structures, and their modular design allows for their functionality to be tailored for specific applications [94]. Both GO and MOFs are often used in powder form, which can limit their applicability due to layer-by-layer agglomeration, pore clogging, hydrophilicity (which complicates the separation from aqueous solutions), and secondary pollution; therefore, they are frequently combined with polymeric materials that act as a supporting matrix, providing the appropriate properties required for fabrication processes such as 3DP, as well as mechanical and chemical stability, reducing agglomeration, and ultimately enabling their practical application in decontamination processes [47,58].

#### 5.1.1. Alginate-Based Materials

Alginate, a naturally occurring, sustainable polysaccharide, particularly in the forms of sodium alginate (SA) and calcium alginate (CA), is widely used in 3D-printed adsorbent materials because of its good printability, gel-forming ability and easy crosslinking in aqueous systems. This biopolymer possesses an intrinsic affinity for metal ions due to the presence of hydroxyl and carboxyl functional groups; therefore, although it is often not the primary adsorbing phase in composite systems, it can still contribute to the overall adsorption performance.

SA was used by Liakos et al. [44] as the main phase to remove Cu^2+^ from water. To form a thermoplastic-based filament that is compatible with FFF, SA was combined with PCL. However, PCL acted only as the carrier matrix, enabling processing and printing. This was validated since PCL alone did not show Cu^2+^’s adsorption ability and the adsorption capacity of the composite increased with an increasing SA content. Therefore, adsorption was attributed to the ion exchange between the sodium in SA (Na^+^) and Cu^2+^, also reporting coordination with carboxylate groups (–COO^−^). The maximum adsorption capacity reached was 93.3 mg·g^−1^, which is an interesting value for an alginate solution; however, it required approximately 30 days to reach equilibrium, which is excessively long for practical applications, and this limits its application in flow-through conditions.

Therefore, most SA applications reported are DIW solutions, where SA was used as a thickener, rheological modifier and structural binder, enabling the fabrication of stable architectures with the incorporation of active adsorbent additives [45,47,57]. Additionally, alginate-based printed networks can provide porous and accessible structures that facilitate metal-ion transport toward the available binding sites [64,68,95].

Zhang et al. [47] used an SA matrix to exploit the adsorption properties of GO, developing a SA/GO monolith to remove Cu^2+^. This adsorbent material could reach a higher adsorption capacity, at 179.32 mg·g^−1^, mainly due to GO’s oxygen functional groups. Additionally, after five cycles of HCl regeneration, these filters were able to retain 74.6% of its initial adsorption capacity. The time required to reach equilibrium was lower but still significant, with the adsorption test running for around 7 h. This research line was followed by using different additives, and it is discussed below in the synthetic polymers subsection (Section 5.1.5).

Wang et al. [95] used a SA/GO ink to generate a CA/GO structure, which enabled the achievement of a hierarchical macroporus structure with a high adsorption capacity of 490.2 mg·g^−1^ and a high selectivity for Pb^2+^ ions. These adsorbents were also regenerated for eight cycles, with a decrease in the adsorption capacity of only 8.26%.

Swathe Sriee et al. [96] paired SA not with GO but with hyaluronic acid, forming a complex for the removal of several metal ions from water, such as Cu^2+^, Cd^2+^, Ni^2+^, Co^2+^, and Fe^3+^. The SA/hyaluronic acid could remove 89.36% of Cu^2+^ and 73.46% of Fe^3+^. Afterwards, the addition of fungal cells further increased the removal capacities to 97.59 and 84.34%, respectively, corresponding to an increase in the order of magnitude of 10 pp., since the fungal cells provided additional binding sites for metal ions.

#### 5.1.2. Cellulose-Based Materials

Cellulose is a natural polymer that has attracted considerable interest for heavy metal removal due to its abundance, renewability, sustainability, and versatility. As a polysaccharide, it possesses numerous hydroxyl groups, providing cellulose with inherent, although limited, adsorption capabilities [42]. These functional groups also allow for chemical modification, functionalizing the material with active groups such as carboxyl (–COOH), amine (–NH_2_), thiol (–SH), and sulfonic acid (–SO_3_H), improving cellulose’s performance as an adsorbent.

Ibebunjo et al. [42] studied a system where cellulose, in the form of microcrystalline cellulose (MCC), was the main adsorbent for the removal of Pb^2+^ from water. For support, PA was used, and it was seen that during printing, MCC and PA were partially sintered together, forming voids and interconnected channels, and enhancing adsorption. After printing, the adsorption capacity of MCC was further improved through functionalization with citric acid, introducing carboxyl groups, and increasing the zeta potential, which led to higher electrostatic interactions and coordination between the adsorbent and the adsorbate. These filters were reused after desorption with HCl, retaining a removal efficiency of 82% in the first cycle and 70% in the fifth. Additionally, the filter was tested for in flow-through column applications. However, its removal efficiency was limited, at a maximum of 19.82 mg·g^−1^.

Rather than acting alone as the primary adsorbent, cellulose, particularly TEMPO-oxidized cellulose nanofibers (TOCNF) [46,50,51,56], often acts as a structural matrix that supports the formation of hierarchical porous architectures and enables the incorporation of additional adsorbent materials [68], such as MOFs, as investigated by Fijol et al. [51] and Abdelhamid et al. [50].

Fijol et al. [51] studied the incorporation of a bioinspired MOF (SU-101) to improve adsorption. With this study, it is possible to evaluate the effect of TOCNFs in adsorption, since adsorbents were tested with and without TOCNF. For support and printability, PLA was used. The addition of TONCF improved the mechanical properties of the adsorbents, maintaining structural integrity after the adsorption and regeneration cycles. However, the highest average Mn^2+^ removal efficiency reported was for acid-activated MOF/PLA filters (53%), while TOCNF/MOF/PLA achieved a slightly lower value of 51%, showing that MOFs have a higher adsorption capacity than TOCNFs.

Abdelhamid et al. [50] developed a binder-free 3DP method based on a cellulose (TOCNF)/ZIF-8 MOF system and developed two materials: one with 2-methyl imidazole (CelloZIF_Hmim) and one with ZnO (CelloZIF_ZnO). The CelloZIF_ZnO showed a higher adsorption capacity than CelloZIF_Hmim. Regarding the best adsorbent, CelloZIF_Hmim showed a good adsorption of Cu^2+^, at 101.2 mg·g^−1^, but lower ability to adsorb Co^2+^ (33.5 mg·g^−1^). Assisted by triethylamine (TEA), the adsorption capacity of Co^2+^ is increased to 108.8 mg·g^−1^. The maximum adsorption capacity reported was higher, at 327 mg·g^−1^ for Co^2+^, via coordination between the ions and the functional groups of the ZIF-8 framework.

#### 5.1.3. Chitosan-Based Materials

Chitosan is a polysaccharide derived from chitin and, like the previously discussed materials, is an abundant and sustainable biopolymer [97], with amine and hydroxyl functional groups along the polymer backbone [98].

Appuhamillage et al. [76] combined the adsorption properties of chitosan with the mechanical strength and printability of diacrylated Pluronic F-127 (DAP) to print hydrogel, which was later UV cured. These filters were able to remove Pb^2+^, Cd^2+^ and Hg^2+^ from water below the detection limits and significantly decrease the Cu^2+^ content of the solution. Additionally, 98% of the Pb^2+^ removal capacity was retained after five regeneration cycles. However, the maximum adsorption capacity is very low, at 0.6 mg·g^−1^.

Aside from its relatively low ion adsorption capacity, chitosan also has low mechanical strength and poor stability in aqueous environments. Therefore, it is also often combined with other polymers, additives, or specific chemical groups, to improve its water stability and adsorption capacity.

Zhang et al. [67] included GO in chitosan, in a system where GO was the main adsorbent and chitosan mainly contributed as a thickener. Due to its predisposition to dissolve in water, a silane coupling agent was introduced to improve its acid resistance, reaching a stable, regeneratable structure, with an adsorption capacity of 269 mg·g^−1^, which can be used for up to six cycles, preserving around 70% of its initial potential. In a following study, Zhang et al. [58] enhanced chitosan/GO adsorption performance by generating porosity through removable SiO_2_, improving the adsorption capacity to 300 mg·g^−1^, while the time required to reach equilibrium decreased by 30 min, to 3 h.

In composites containing GO or MOFs, these additives typically serve as the primary adsorbents, while chitosan facilitates the composite formation, printability, and contributes additional adsorption sites, similarly to alginate and cellulose.

Joseph et al. [99] paired chitosan with MOFs, using a bimetallic Mn-doped MIL-100(Fe)-based MOF/chitosan composite. For structural support, PS was added. The best formulation could remove 99% of As^5+^ and 95% of As^3+^ from water, with an adsorption capacity of 21.1 mg·g^−1^ and 30.2 mg·g^−1^, respectively. The study also demonstrated that SLS is an effective strategy for transforming fragile MOF/chitosan powders into mechanically stable and functional filters for As^3+^/As^5+^ removal.

Wu et al. [63] combined chitosan with the ZIF-67 MOF and printed three types of filters: ZIF-67 (3D-ZIF-67), chitosan (3D-CS), and the composite material (3D-CS-ZIF-67). This study demonstrated that although ZIF-67 provides the primary adsorption capacity, the 3D-CS–ZIF-67 composite exhibited superior overall performance due to the synergistic combination of adsorption capability and mechanical properties. Additionally, the competing ions influenced Pb^2+^ adsorption differently, with Cd^2+^ reducing adsorption and Cu^2+^ enhancing it through ion-exchange processes involving the release of Co^2+^ ions from ZIF-67, generating additional N-containing active sites that promote Pb^2+^ adsorption, as well as through the reduction in mass transfer resistance.

#### 5.1.4. Other Biopolymer-Based Materials

Some authors have combined cellulose and alginate, as their complementary rheological properties and functional groups enable the fabrication of structured adsorbents with improved printability and adsorption performance, combining cellulose’s ability to create hierarchical pore structures with the rheological properties of SA.

Thakare et al. [93] fabricated alginate/methylcellulose hydrogels by DIW, with and without immobilized algae cells. The printed filters reduced the Cu^2+^ concentrations to below detection standards (99.97 to 100%). However, the addition of algae reduces the treatment time by one third, demonstrating the benefit of integrating biological components to enhance efficiency.

Wu et al. [43] also combined the two materials through an alginate/cellulose nanocrystals (CNC) mixture, crosslinked with a CaCl_2_ solution. These adsorbents were able to achieve 97.22 mg·g^−1^ of Cu^2+^ removal, and it was also effective for other metals. Additionally, they could be regenerated, retaining 88% of their adsorption capabilities after five cycles.

Abdelhamid et al. [46] developed TOCNF/SA materials (CelloCOF) incorporating two different covalent–organic frameworks (COF). CelloCOF materials reached a very high maximum removal capacity for Cu^2+^ (410.8 mg·g^−1^), which was attributed to coordination with nitrogen-containing functional groups. The selectivity was shown for Fe^3+^. The materials retained their structure and adsorption capacity after at least three regeneration cycles using HCl.

Finally, Zhang et al. [68] developed a 3D-printed absorbent using 2,3-dimercaptosuccinic acid (DMSA)-modified GO dispersed in cellulose, with SA to improve printability, as in the previous cases. The sulfhydryl (–SH) groups from DMSA created abundant active sites for metal binding, achieving a maximum Cu^2+^ adsorption capacity of 250 mg·g^−1^, mainly due to chelation. Column experiments showed that the material’s potential for practical water treatment applications, and regeneration, retaining around 80% of the adsorption capacity was possible for five cycles.

Other biopolymers, such as chitin, have also been explored. Chitin is a natural polysaccharide found in crustaceans, insects, and fungal cell walls [97], and it enables adsorption due to functional groups such as acetamide (–NHCOCH_3_) and hydroxyl (–OH).

Fijol et al. [56] showed that reinforcing PLA with chitin (ChNF/PLA) and TOCNF (TOCNF/PLA) significantly enhances the adsorption capacity compared with pure PLA, with TOCNF providing the greatest improvement due to the presence of carboxylate and carboxyl groups from TEMPO oxidation, compared to the amino sugars and hydroxyl groups in chitin. However, the materials exhibited limited reusability, with the adsorption capacity decreasing substantially after repeated regeneration cycles, with the biopolymers retaining only 50% of their adsorption capacity in the second cycle and 30% in the third.

Protein-based materials have also been considered for removal component applications due to their rich surface chemistry, containing functional groups such as amine (–NH_2_), carboxyl (–COOH), hydroxyl (–OH), and thiol (–SH), and showing their ability to produce hydrogels and aerogels. Among the protein-based materials used are gelatin and bovine serum albumin (BSA).

The incorporation of functional nanofillers and amine-rich polymers into gelatin/SA-based systems enhanced both the structural performance and adsorption efficiency. Particularly, the incorporation of PEI, a polymer with a high density of amine groups, by Finny et al. [57], reached an excellent Cu^2+^ adsorption capacity of 633.2 mg·g^−1^, taking into consideration the reference mass of the dry material, and 43.42 mg·g^−1^ if the hydrated mass is considered, highlighting the critical role of amine functionality in complexation mechanisms. Montmorillonite nanosheets (MMTNs), incorporated by Miao et al. [64], enabled the adsorption of 134 mg·g^−1^ of Pb^2+^ due to chemisorption in the MMTNs and the stable interpenetrating polymer network with abundant macropores and lamellar structures generated, which facilitates the transport of metal ions.

Masud et al. [53] formulated a graphene-biopolymer aerogel, using PDA and BSA as bio-inspired matrices to provide the rheological properties required for DIW and improve structural stability in aqueous media. PDA and BSA contributed to contaminant removal, while graphene was the main adsorbent phase. The resulting hydrogel-derived aerogel remained stable in water and achieved adsorption capacities of up to 45.05 mg·g^−1^ for Cr^6+^.

#### 5.1.5. Synthetic Polymer-Based Materials

In addition to biopolymers, several synthetic polymers have been explored as components of 3D-printed adsorbents, some of which were previously discussed when combined with biopolymers. Among the most common are PLA, PVA, TPU, and polyacrylic acid (PAA).

PLA is a bio-based synthetic thermoplastic, which is typically produced from renewable resources. It is widely adopted in 3DP as an alternative to petroleum-based polymers, since it has good mechanical properties, stability, and ease of processing. Overall, PLA-based structures demonstrate good mechanical stability and processability, where their adsorption performance is governed by the incorporated active phases (such as hydroxyapatite (HAp) and GO).

Fijol et al. [100] incorporated HAp, with uniform pore geometry, inter-connected channels and the homogeneous distribution of HAp, exhibiting good adsorption performance for Pb^2+^ and Cd^2+^, reaching a maximum capacity of 360 mg·g^−1^, and showing promising properties for scalable water treatment applications. Wu et al. [101] used the same materials, also pairing them with chitosan. The combination of 3DP and freeze drying enabled it to create a hierarchical macro- and microporous structure, still reaching a promising, although lower, capacity of 119 mg·g^−1^ for Cu^2+^.

Park et al. [55] included GO, removing 95.86% of Cd^2+^ ions from water and showing strong long-term performance when operating the fixed-bed column with scalability possibilities, and retention of around 70% of the adsorption properties after five cycles.

Another very commonly used synthetic polymer is PVA. PVA is a water-soluble polymer, rich in hydroxyl groups (–OH), produced through the hydrolysis of polyvinyl acetate. There are two main approaches followed for using PVA: (i) utilizing its film-forming capability to produce hydrogels after cross-linking, enabling the material to swell in water rather than dissolve, or (ii) exploiting its water solubility to generate porosity, increasing the availability of adsorption sites, decreasing the density and creating stronger hierarchical roughness.

Following the first path, Asghartabar Kashi et al. [62] developed a PVA/PAA hydrogel which revealed very high adsorption capability for Pb^2+^, reaching an adsorption capacity of 896 mg·g^−1^ in 2 min. Crosslinking was achieved using a photosensitizer, whose increasing concentration led to a higher crosslinking density, resulting in a stronger polymer network, improved mechanical strength and stiffness, and enhanced printability.

Zhang et al. [45], building on a previous study using GO/SA [47], incorporated PVA into the composite to create a soft physically crosslinked network, increasing the mechanical properties of the hydrogel (retaining its structure after 70% deformation) and its adsorption capacity from 179.32 to 208 mg·g^−1^.

Lan et al. [102] and Kanaan and Piedade [103] both explored PVA/TPU blends, although from two distinct perspectives.

Lan et al. [102] printed an adsorbent from a commercially available PVA/TPU blend and, after printing, crosslinked the PVA with glutaraldehyde in the presence of HCl, forming a hydrogel. Afterwards, *Chlorella pyrenoidosa* was loaded into the printed cap as the primary Pb^2+^ adsorbent, removing ions through interactions with its hydroxyl and phosphoryl groups. The system achieved a removal efficiency of 75.61% and maintained similar performance after seven regeneration cycles.

Kanaan and Piedade [103] used the same commercially available TPU/PVA but followed the second path for the use of PVA, and, after printing, washed the components to remove the PVA, leaving a TPU-dominated structure which was able to preserve the shape and structural integrity of the printed part. Additionally, its swelling characteristics made the adsorption sites even more accessible. These filters were able to remove more than 80% of ions from water, including Fe^3+^, Cu^2+^ and Zn^2+^.

Other polymers, such as PAA, ABS, and PEGDA, were also explored. Shahbazi et al. [75] used an aginate/clay-g-PAA system and was able to reach excellent Pb^2+^ adsorption: 532 mg·g^−1^ after 5 min. The addition of PAA adds the carboxyl and carboxylate groups to the SA backbone after grafting, improving adsorption. Nanoclay, apart from its adsorbing properties, contributes to the mechanical properties and stability of the final material.

Ji et al. [104] used an ABS-based polymer to test the hypothesis that with controlled hydrolysis of nitrile groups, the ABS surface would increase metal ion adsorption, showing that increasing from 5 to 15 min of hydrolysis, the Co^2+^ removal increased from 8.8 to 12.4%, due to the generation of carboxylate and amide groups, increasing the hydrophilicity of the material. However, the biggest change was felt when polyoxometalate (POM) anions were used to functionalize the ABS surface, which, due to its high negative charge ([α-PW_9_O_34_]^9−^), was able to completely remove the Co^2+^.

Burratti et al. [105] used PEGDA hydrogels doped with silver nanoparticles, printed with a custom printer for Hg^2+^ removal. The filters showed removal efficiencies of 86–94%. However, their adsorption capacity is still low, at 0.55–0.61 mg·g^−1^, with long contact times of 8 h.

#### 5.1.6. Comparison Between Studies

**Figure 8:**
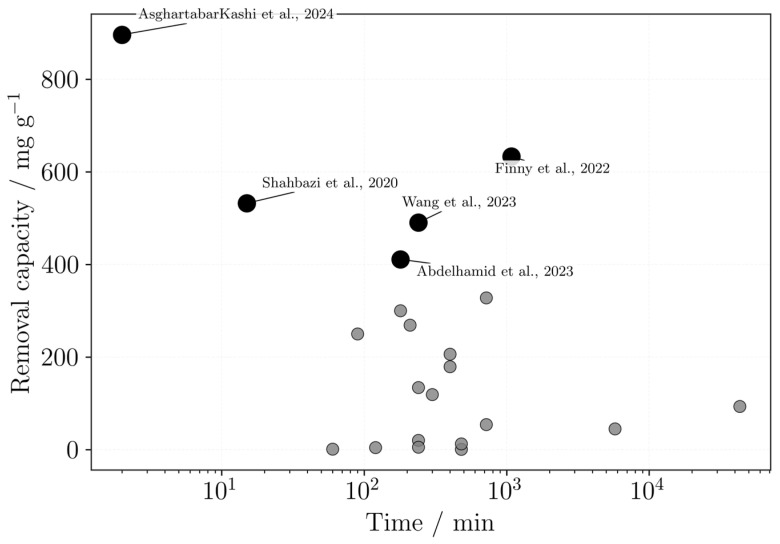
Removal capacity comparison for heavy metal removal from water studies, highlighting Abdelhamid et al. [46], Finny et al. [57], Asghartabar Kashi et al. [62], Shahbazi et al. [75], and Wang et al. [95].

Based on the studies reporting maximum adsorption capacities and times to equilibrium, a comparative plot was generated (Figure 8), highlighting cases where high capacities were achieved at relatively short contact times. However, this comparison is based only on the reported values and should not be interpreted as a direct comparison, since the results may vary depending on the experimental conditions used in each study. When the equilibrium time was not given, the experiment time was considered.

The best cases highlighted are from Asghartabar Kashi et al. [62], Shahbazi et al. [75], Finny et al. [57], Wang et al. [95], and Abdelhamid et al. [46]. All these studies used DIW processes, which are clearly stated by Wang et al. [95] and Abdelhamid et al. [46]. Most materials used alginate in their formulation, with Shahbazi et al. [75] pairing it with PAA and nanoclay, Finny et al. [57] with gelatin and PEI, Wang et al. [95] with GO, and Abdelhamid et al. [46] et al. with TOCNF and COF. However, the most promising ones are from Asghartabar Kashi et al. [62], followed by Shahbazi et al. [75] and Abdelhamid et al. [46], due to their lower equilibrium times of 2, 15 and 180 min, respectively.

### 5.2. Dye Removal

**Figure 9:**
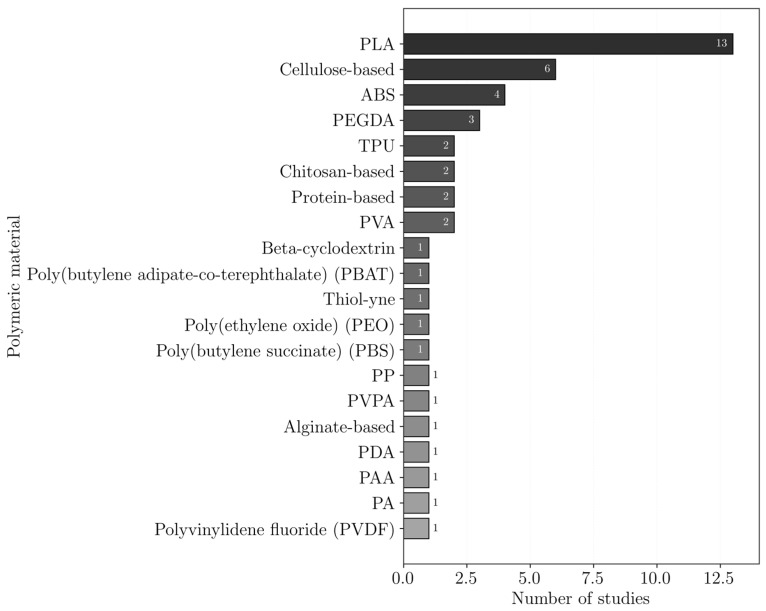
Polymers reported in the dye contamination reviewed studies.

The polymers used in dye removal studies are shown in Figure 9. In this analysis, polymers are not categorized based on their function within the system (e.g., as structural matrices or as additives). Instead, all polymers reported across the reviewed studies are considered collectively. The distribution highlights a noticeable tendency toward the use of PLA supports.

**Figure 10:**
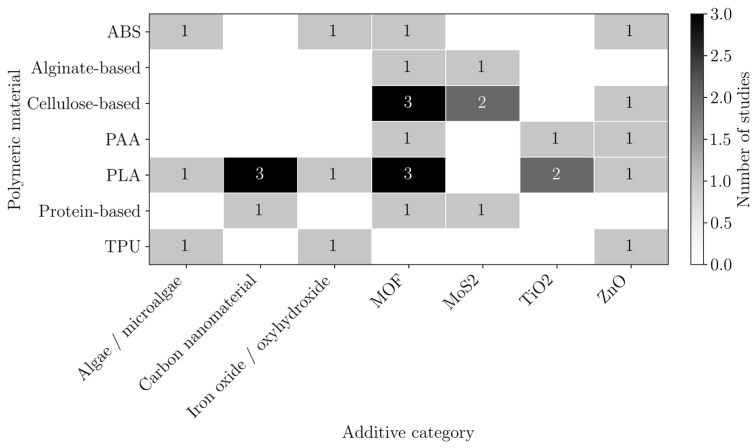
Polymers and additives reported in the dye contamination reviewed studies.

In several studies, polymers are further incorporated with additives possessing high adsorption affinity or degradation properties to improve the overall performance, as shown in Figure 10. The most used additives are MOFs; carbon nanomaterials, particularly GO; and MoS_2_.

#### 5.2.1. Cellulose-Based Materials

Cellulose-based materials have been used to adsorb both cationic and anionic dyes, since their versatile surface chemistry allows for chemical modification, enabling the surface to be tailored to carry either positive or negative charges.

On the one hand, Ranjan et al. [49] used a negatively charged phosphorylated cellulose gel to remove the cationic dye Methylene Blue, achieving 99% removal and regeneration over at least seven cycles, although the desorption efficiency decreased with reuse. The material was synthesized via an environmentally preferable process compared to conventional TEMPO oxidation, according to life cycle assessment. Subsequent modification through the in situ growth of MoS_2_ [80] produced filters with comparable dye removal efficiency, reusability for at least five cycles, and added antimicrobial activity. Adsorption in both systems was driven by negatively charged phosphate groups, while MoS_2_ additionally enabled the photocatalytic degradation of Methylene Blue under light irradiation.

On the other hand, Shojaeiarani et al. [106] developed a cellulose-based hydrogel, using CNC, with PEO to improve printability and mechanical stability. CNC were incorporated to enable the adsorption of both a cationic dye and an anionic dye. To enable the adsorption of each of the dyes, the CNCs were chemically modified to obtain either cationic or anionic forms, to capture anionic and cationic dyes, respectively. Then, these materials were used to create a multilayered hydrogel material with oppositely charged layers, capable of adsorbing both cationic (95.23%) and anionic dyes (75%). The simultaneous adsorption of oppositely charged dyes and the limited interaction between layers can be attributed to the mobility of dye molecules, which diffused through the porous hydrogel to reach adsorption sites, while the oppositely charged CNCs were immobilized within the hydrogel, preventing direct interaction between layers. However, in this work, Methylene Blue was treated as the cationic dye and Malachite Green as the anionic dye, although Malachite Green is typically cationic. This may have reduced the charge difference between the adsorbent and adsorbate, potentially contributing to its weaker adsorption. Challenges also remain regarding regeneration and recyclability.

The adsorption capacities of cellulose (TOCNF) and MOFs were compared by Fijol et al. [51], since they removed Methylene Blue through adsorption, using PLA-supported MOF adsorbents with and without TOCNF. The MOF/PLA filters removed a higher dye content (52%) than TOCNF/MOF/PLA (43%), which shows that MOF has better adsorption properties. Therefore, as for heavy metals, cellulose was also mainly explored as a platform to integrate more active adsorbent components, such as MOFs.

Abdelhamid et al. [50] took advantage of both, combining cellulose and MOF (ZIF-8) to adsorb cationic, Rhodamine B, and anionic Methyl Blue dyes, combining the material with two different additives: ZnO (CelloZIF_ZnO) and Hmim (CelloZIF_Hmim). Although these adsorbents had previously demonstrated excellent performance toward positively charged heavy metal ions, with a maximum adsorption capacity of 327 mg·g^−1^, they showed a markedly greater affinity for the anionic dye than for the cationic one (98% of removal efficiency compared to negligible adsorption). The weak adsorption of Rhodamine B was overcome through catalytic degradation with NaBH_4_, achieving 85% removal. This behavior may appear to be counterintuitive, as the system had shown good adsorption of positively charged metal ions. However, in that part of the study, the material already showed a clear preference for Cu^2+^ (101.2 mg·g^−1^) over Co^2+^ (33.5 mg·g^−1^), only increasing Co^2+^ adsorption after the addition of TEA, which promotes deprotonation within the adsorbent. Additionally, this metal ion adsorption was attributed to coordination interactions, while dye adsorption was generally associated with interactions at the MOF surface, possibly involving the Zn^2+^ nodes. These findings underline the importance of understanding selectivity when designing multifunctional adsorbents.

#### 5.2.2. Other Biopolymers

Other biopolymers, such as chitosan, alginate, and gelatin, have also been investigated for dye removal, although challenges related to mechanical stability, adsorption efficiency, and regeneration remain, due to their relatively weak structural networks and susceptibility to degradation and gradual loss of adsorption sites during repeated use.

Yusoff et al. [107,108] developed a chitosan-based structure supported by PEGDA for the adsorption of Methyl Orange from water. Since Methyl Orange is an anionic dye, effective adsorption requires a positively charged adsorbent surface. This was achieved under acidic conditions, where the amine groups of chitosan become protonated. PEGDA itself exhibits a very low adsorption capacity; therefore, it can be concluded that the adsorption performance mainly originates from chitosan. Under optimal conditions, the material reached a maximum adsorption capacity of 12.7 mg·g^−1^ and could be reused for at least four cycles, maintaining about 78% of its initial efficiency.

Alves et al. [109] studied a hydrogel composed of alginate, carboxymethylcellulose and gelatin, with and without MOF particles, and with and without catalytic particles (MoS_2_). The MOF particles were intended to provide adsorption sites and form a hetero junction with MoS_2_, while MoS_2_ acted as a photocatalyst facilitating electron transfer. The addition of 7.5% MOF/MoS_2_ increased the adsorption of Methylene Blue from 17% to 96%, although equilibrium was reached only after long contact times (24 h). When photocatalytic activity was considered, a removal efficiency of 89% was achieved in about 7 h. However, although increasing the MOF/MoS_2_ content increased the removal efficiency, it weakened the mechanical stability of the aerogel and reduced the regeneration efficiency, as the adsorption capacity retention decreased with higher loading. Furthermore, the composites exhibited structural fracturing after reuse cycles, indicating lower durability compared to the pristine hydrogel matrix, highlighting that, although most studies discussed were based on the incorporation of additives, this can heavily penalize the durability and regeneration capacity of the adsorbent.

#### 5.2.3. PLA-Based Materials

PLA, similar to its use in heavy metal removal, is mainly used as a 3D-printed structural support which enables the incorporation of the active components that are responsible for dye adsorption or degradation.

Most PLA-based systems reported for dye removal rely primarily on adsorption mechanisms governed by surface charge interactions, particularly the attraction between negatively charged surfaces and cationic dyes. For instance, Shi et al. [110] used the support of PLA to print films functionalized with in situ growth of Cu-MOFs for Malachite Green removal. The filters achieved over 90% removal within 10 min across different water sources, showing their suitability for scalable applications in flow-through systems. Good reusability was observed, retaining around 90% adsorption capacity after three cycles and around 70% after five cycles.

Park et al. [111] also used PLA as a support material: in this case, for GO. The adsorbent surface was further modified to enhance GO incorporation. The treatment with 95% acetone generated new hydrophilic functional groups, such as carboxylic acid and hydroxyl groups, through the cleavage of ester bonds in the aliphatic polyester. As a result, the negatively charged filters were able to adsorb approximately 75% of the Methylene Blue cationic dye.

The reinforced PLA studies presented until this point are based on the principle of negatively charged surfaces adsorbing cationic dyes; Delikanli et al. [92] compared the adsorption of cationic and anionic dyes using the same adsorbent, functionalized PLA reinforced with wood. The activation of neat PLA introduced functional groups and surface porosity, enabling up to 45.3% removal of the cationic dye Crystal Violet, which was further enhanced by the addition of wood through electrostatic, hydrogen-bonding, and π–π interactions. In contrast, removal of the anionic Congo Red was low (9.21%) due to electrostatic repulsion, showing a high selectivity of these components towards positively charged dyes. The filters maintained about 95.8% of their initial capacity after three cycles, decreasing to 51.4% after seven cycles.

Because adsorption systems often show selectivity toward specific dye charges and may require long equilibrium times, several studies have incorporated complementary mechanisms, particularly photocatalytic degradation, to broaden applicability and improve performance. An example is the study by Dhillon et al. [79], who used a BiFeO_3_-coated PLA catalyst to remove the anionic dye Congo Red and the cationic dye Methylene Blue from water. Both dyes were degraded via photocatalysis; however, Congo Red showed a higher removal efficiency (98.9%) than Methylene Blue (74.3%), which was due to Congo Red containing azo bonds, which are very susceptible to oxidative attack, and Methylene Blue being much more chemically stable. A machine learning (ML) model was used to predict dye removal at different times and excitation wavelengths, showing good correlation with experimental results and demonstrating its ability to capture complex linear and nonlinear relationships between input and target variables. The catalyst could be used for five cycles, maintaining 97% of its removal capability.

Ortega-Columbrans et al. [81] studied a TiO_2_-doped PLA. Dye removal was described as involving an initial adsorption step, followed by photocatalytic degradation under UV irradiation by the TiO_2_ particles dispersed in the PLA matrix. The highest removal efficiency for Methyl Orange (anionic) was 98% after 24 h of irradiation. However, this value was obtained for a cast-like sample, rather than a 3D-printed part, with the higher efficiency attributed to the greater porosity of the cast structure. For the 3DP filament, the maximum removal efficiency reached was 90%, with 80% for the 3D-printed parts.

Cirillo et al. [86,87] reported two consecutive studies in which PLA/carbon black filaments were further functionalized with metallic particles and then electrochemically activated for dye removal. In the first study [86], silver particles were incorporated and Methyl Orange, an anionic azo dye, was used as the target contaminant in a chloride-containing electrolyte. After the optimization of the operating conditions, complete dye removal was achieved through electrochemical degradation. In the second study [87], copper nanoparticles were used instead, and Methylene Blue was selected as the target contaminant. Although the overall approach was similar, replacing silver with copper promoted stronger surface-bound hydroxyl radical chemistry. The removal efficiency was slightly lower, reaching about 97%, which may be related to the greater resistance of Methylene Blue to degradation compared with azo dyes, as also noted by Dhillon et al. [79]. Both systems also showed good reusability, with the silver-based filter maintaining its performance over five cycles and the copper-based filter over ten cycles.

Another approach for removing anionic dyes using negatively charged polymeric materials is electrostatic repulsion. Pereira et al. [112] developed a PLA-based ultrafiltration membrane for the removal of anionic dyes such as Methyl Red, Indigo Carmine, and Reactive Black 5. Dye rejection resulted from a combination of electrostatic repulsion from the negatively charged membrane surface and size exclusion through pores of 4–7 nm. The system achieved removal efficiencies of up to 98% and showed good regeneration, with improved performance under alkaline conditions due to the increasingly negative zeta potential of the membrane surface.

#### 5.2.4. Other Polymer-Based Materials

*Adsorption-based systems*

The main adsorption target addressed in the reported studies was cationic Methylene Blue. Wang et al. [113] printed ABS-supported MOF Cu-BTC coated parts, achieving a maximum removal efficiency of 93% within 30 min. Adsorption was attributed to physical phenomena and interactions between the nitrogen atoms of Methylene Blue and the copper sites of the Cu-BTC coating. Accordingly, increasing the number of coating layers enhanced the adsorption capacity. However, this approach remains limited by its surface-coating nature, and it does not exploit the creation of adsorption sites throughout the bulk porous structure. Five different MOF fillers were explored by Li et al. [114] on PA-12 films, concluding that NH_2_-MIL-101(Al) was the best additive to use for adsorption studies, due to its thermal stability, high surface area and high adsorption capacity (222 mg·g^−1^). It was concluded that the dye adsorption capabilities of the MOF were enabled by the PLA support, since the adsorption capacity of the PLA/MOF structure was 152 mg·g^−1^.

Xia et al. [115] and Zheng et al. [116] took advantage of the adsorption properties of biomass, also using PLA support. On the one hand, Xia et al. [115] developed an adsorbent material composed of PLA, PBAT, and *Chlorella pyrenoidosa* microalgae. The inclusion of 30% of microalgae increased the adsorption from 0.06% to 92.66%, indicating that the microalgae were the main adsorbent, due to the higher number of functional groups and adsorption sites. The filters also demonstrated reusability, retaining 72% of their adsorption efficiency after six regeneration cycles.

On the other hand, Zheng et al. [116] also proposed a biomass-based approach, but developed an adsorbent composed of PLA, PBS, and PVA doped with Camellia seed powder. As in the previous study, the polymers mainly have a support role, aside from PVA, which, as in the case of Kanaan and Piedade [103], is used as a sacrificial porogen, increasing the material porosity from 21.24 to 48.37%. The main adsorbent is, therefore, the Camellia seed powder, since it contains hydroxyl, amine, carbonyl and aromatic groups. The dye removal efficiency of these filters was almost 100%, retaining 99% of its removal efficiency after seven cycles.

Custom polymeric solutions were also developed for dye adsorption for Rhodamine B and Malachite Green, as the material from Ng et al. [117], a photocurable PEGDA and 2-hydroxylethyl methacrylate (HEMA) polymer matrix (PEGDA: HEMA), which was compared to pure PEGDA for the removal of Rhodamine B. It was observed that the PEGDA: HEMA polymer almost doubled the maximum adsorption of the PEDGA, due to the introduction of hydroxyl groups, and reached a maximum adsorption of 94.86%. Shahzadi et al. [118] formulated a material based on thiol and alkyne monomers for the adsorption of Malachite Green. The material was printed as a tropophobia ball, which showed that the material exhibited good printability and resolution capability. Additionally, it exhibited excellent removal of Malachite Green from water, removing 100% of the material in 10 min for six consecutive cycles, with a maximum adsorption capability of 588 mg·g^−1^. These promising adsorption properties are attributed to the hydroxyl (–OH) and thiol (–SH) groups on the adsorbent’s surface.

*Degradation-based systems*

Some authors also propose degradation processes as a path for the removal of both anionic and cationic dyes, which is the most common photochemical degradation. Li et al. [119] developed PLA fractal structures, using different plasma grafting methods and ZnO and TiO_2_ photocatalyst particles. From the diverse configurations and material combinations used, the best performance for Rhodamine B removal (94%) was obtained by ZnO particles and PAA grafting agent, preserving around 90% of the removal efficiency after three regeneration cycles. Removal occurred mainly due to photocatalytic degradation with simulated sunlight; therefore, the ZnO particles had a significant responsibility for the outcome. Wang et al. [120] continued this research line, by continuing to use plasma to modify surfaces and a similar geometry to degrade dyes by catalysis. However, this second work focused on Zn-doped CdS catalysts, and the grafting changed to a PVPA coating, a method which had already been preliminarily attempted in the first work. These filters also enable regeneration, with Zn-doped CdS showing better stability than undoped CdS.

Also, based on photochemical degradation, Zhang et al. [121] prepared ABS/TPU/CaSiO_3_ skeletons for the removal of Rhodamine B from water. The introduction of ZnO nanospheres to the skeleton surface, as shown by [119], increased the adsorption sites and the removal efficiency from 76% to 98%, shortening the treatment time from 48 to 8h. The ABS/TPU/CaSiO_3_ skeleton primarily acted as a support, concentrating Rhodamine B near the surface, while ZnO was the photocatalyst, under UV irradiation. This created a synergistic removal mechanism in which the skeleton enhanced the dye concentration near ZnO, while ZnO promoted dye degradation. In a following study within the same research line, Liu et al. [122] used the ABS/TPU skeletons to degrade Methyl Orange. In this case, the CaSiO_3_ and ZnO were replaced by *chlorella*-assisted Fe_2_O_3_. *Chlorella* not only provided active sites for Fe_2_O_3_ to attach but also participated in the degradation. This new system enabled the light irradiation to change from UV to visible light. In this case, since the dye contaminant was anionic, the adsorption component of the removal was less significant (31%, compared to 76% for the cationic dye), while the photocatalysis gained importance, reaching a total removal of 91%.

Fenton degradation can also be used for dye degradation, changing the activation of the oxidation process from light to a reaction with ferrous ions (Fe^2+^). Therefore, D’Accolti et al. [82] used iron oxide-coated PLA scaffolds as a heterogeneous catalyst for Fenton degradation of Methylene Blue. In the presence of hydrogen peroxide, at low pH, hydroxyl radicals are generated, oxidizing dye, and achieving complete removal in under 30 min, which was shown to be particularly difficult for Methylene Blue by previous studies, due to the absence of azo bonds.

Also on degradation, Bößl et al. [88] tried to develop a piezocatalytic system for Rhodamine B, combining a piezoelectric support (PVDF) with the piezocatalytic properties of BaTiO_3_. However, the piezoelectric degradation results were not as promising as expected, and pure PVDF showed better performance than the composite. This better performance was attributed to sonoadsorption and adsorption-enhanced degradation. Dye molecules adsorbed onto PVDF through hydrophobic interactions, as well as π–π and van der Waals interactions, together with porous adsorption, while ultrasound enhances the mass transfer. As a result, radicals formed near the surface, where dye molecules are already concentrated, had a higher probability of reacting, thereby increasing the efficiency of sonochemical degradation.

Other types of oxidative degradation have also proven to be effective in dye degradation. Zheng et al. [89] developed PP-shell/ABS-core filaments for the removal of Methylene Blue and Crystal Violet from water. After printing, the ABS core was dissolved with acetone, creating hollow channels. The PP shell was then functionalized with sulfuric acid, introducing sulfonic acid groups that became negatively charged in water and enabled the adsorption of cationic dyes, with a maximum capacity of 138 mg·g^−1^. For anionic dyes such as Acid Orange 7, adsorption was weak due to electrostatic repulsion. In this case, potassium persulfate was added to promote degradation, generating reactive SO_4_^•–^ radicals through interaction with the sulfonated fibers, which break the dye molecules, particularly at the azo-bond, achieving about 95% removal.

#### 5.2.5. Comparison Between Studies

**Figure 11:**
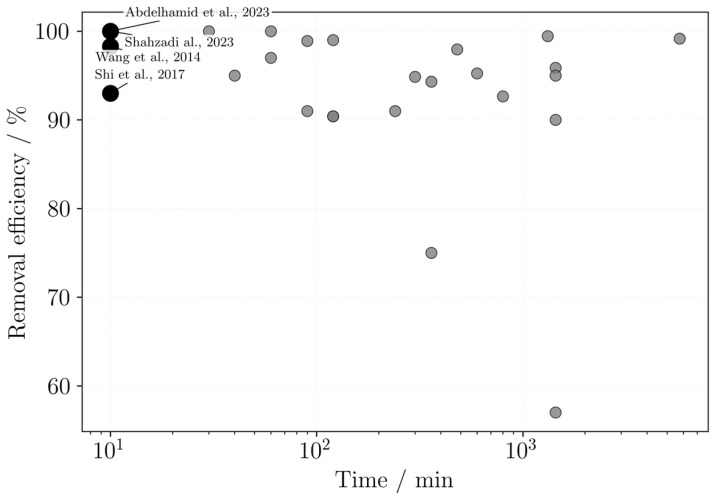
Removal capacity comparison for dye removal from water studies, highlighting Abdelhamid et al. [50], Shi et al. [110], Wang et al. [113], and Shahbazi et al. [118].

A comparative analysis was performed using the reported removal efficiencies together with the corresponding equilibrium times, and the results are presented in Figure 11. The plot allows for a visualization of systems that combine relatively high efficiencies with shorter contact times. It should be emphasized that this representation is based solely on the values reported in the literature and therefore does not constitute a direct comparison between studies, as experimental conditions, materials, and methodologies differ. Additionally, reporting only the removal efficiency makes the results strongly dependent on the initial dye concentration used; for instance, when the dye concentration in the solution is lower, complete removal can be achieved, even with a lower adsorption capacity. A comparison based on adsorption capacity would be more informative; however, this was not possible because many studies did not report the capacity values. In cases in which the equilibrium time was not explicitly reported, the total experimental duration was used instead.

Most studies report high removal efficiencies. Therefore, the most promising results were distinguished mainly by the time required to achieve them. The fastest performances were reported by Abdelhamid et al. [50] and Shahbazi et al. [118], both of which reached 100% removal within 10 min, followed by Wang et al. [113], who achieved 98.3%, and Shi et al. [110], who reported 93% in the same time frame. In all these studies, adsorption was the main removal mechanism. Abdelhamid et al. [50] also applied photocatalytic degradation; however, adsorption alone already led to 98% removal, while the combined process increased the removal efficiency to the final value of 100%. Additionally, both Shahbazi et al. [118] and Shi et al. [110] used Malachite Green as a contaminant, showing that, for this contaminant, the solution by Shahbazi et al. [118] appears to be more promising. However, the subjectivity of this analysis is proven by [118] and Wang et al. [113], which report both removal efficiencies and adsorption capacities, since the maximum efficiency capacity reports are 100 and 98.3%, and the maximum adsorption capacities are 588 and 64.3 mg·g^−1^.

## 6. Geometric Design of 3D-Printed Polymer-Based Treatment Systems

**Figure 12:**
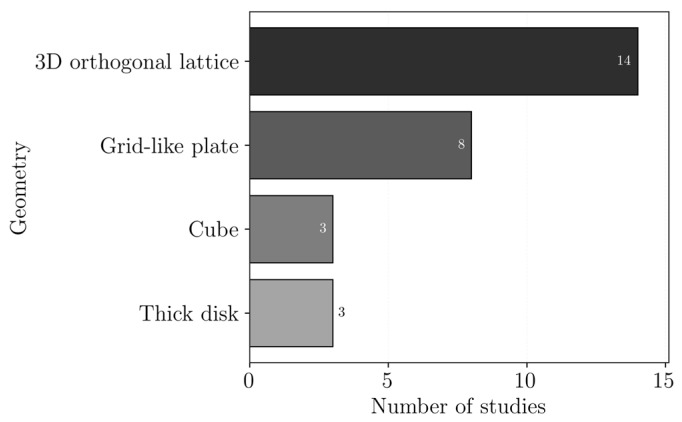
Most common geometries reported in the reviewed studies.

The most common 3D-printed geometries used for the removal components can be found in Figure 12. Geometries that were reported in only one study were excluded from the plot.

**Figure 13:**
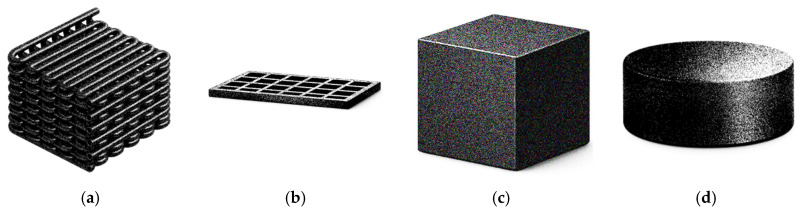
Representative geometries commonly reported for 3D-printed components used in water treatment applications: (**a**) 3D orthogonal lattices, (**b**) grid-like plates, (**c**) cube, and (**d**) thick disks.

The four most common geometries are 3D orthogonal lattices (Figure 13a), grid-like plates (Figure 13b), cubes (Figure 13c), and thick disks (Figure 13d). These relatively simple geometries are often used primarily to evaluate the performance of different material combinations. Lattice or grid structures are typically employed to exploit the macroporosity enabled by 3DP, in addition to the meso- and microporosity that are inherent to the material, creating a hierarchical porosity structure. All these geometries were applied in adsorption and photodegradation processes, whereas more dense structures, such as 3D orthogonal lattices and thick disks, were additionally used for filtration applications.

However, more complex geometries have also been explored, and comparative studies are discussed in the following sections.

### 6.1. Heavy Metal Removal

To evaluate whether 3DP offers advantages over conventional manufacturing techniques, several authors compared 3D-printed structures with their bulk counterparts. Wu et al. [43], for example, fabricated 3D orthogonal lattice structures composed of hollow filaments using alginate and CNC and compared their performance with that of a solid block made from the same hydrogel for Cu^2+^ removal. The porous printed construct exhibited a higher adsorption capacity, reaching about 67.6 mg·g^−1^, compared with 47.8 mg·g^−1^ of the hydrogel. The adsorption rate also increased by 3.14 times. After printing, the constructs were freeze-dried to generate a secondary pore structure that combines macropores from the printed geometry with micro- and mesopores created during freeze-drying. This hierarchical porosity increases the exposure of adsorption sites and shortens ion diffusion pathways, highlighting the importance of multiscale porous architectures in improving the adsorption performance. Swathe Sriee et al. [96] also compared printed hydrogel patches and beads of alginate/HA/fungal systems and concluded that the 3D-printed patches had an increase in removal efficiency of 20–25% due to their higher surface area and porosity. In a similar study, Rezanavaz et al. [123] compared the adsorption capacity of a 3D-printed molecularly imprinted polymer’s (MIP) geometry against its UV-polymerized bulk counterpart, showing that the 3D-printed material had around a 10 times higher adsorption capacity, due to its more organized and hierarchical microporous structure and increased surface area.

**Figure 14:**
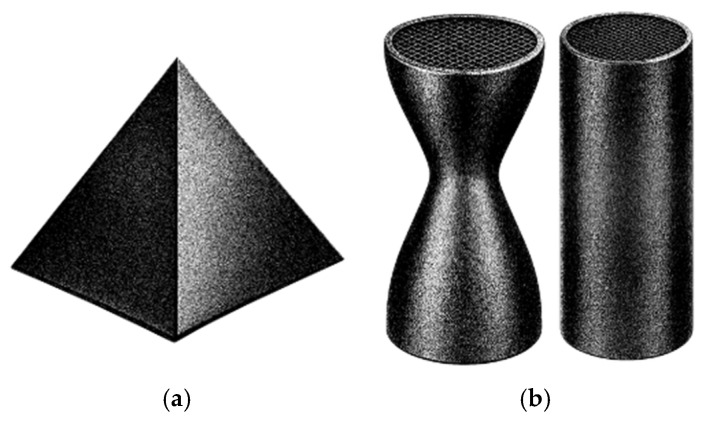
Representative geometries used by (**a**) Kanaan and Piedade (adapted from [103]) and (**b**) Fijol et al. (adapted from [56]).

Kanaan and Piedade [103] preliminarily tested semi-spherical and pyramidal structures, finding that pyramidal geometries (Figure 14a) create a more hierarchical surface roughness and greater surface exposure compared with semi-spheres. This configuration facilitates water penetration and increases the contact area between the material and the solution, while also promoting greater diffusion of contaminants into the pores. Consequently, the pyramidal structure was selected for the adsorption experiments.

This study also exploited water-soluble PVA as a sacrificial component to create a hierarchical porous structure. Together with other studies, it demonstrates that a feature often perceived as a limitation of 3DP, the formation of porous structures, can instead represent a significant advantage for water treatment applications. The inherent porosity of 3D-printed materials can enhance the contaminant adsorption by increasing the accessible surface area and improving mass transfer within the structure [58,63,96,103].

Looking into the geometry of the 3D-printed parts, Fijol et al. [56] printed PLA reinforced with ChNF (ChNF/PLA) and TOCNFs (TOCNF/PLA) in both cylindrical and hourglass geometries (Figure 14b). In addition to the overall shape, different pore architectures were also investigated, including structures with uniform pore sizes and structures with a gradient in pore size. All structures presented improved mechanical properties when compared to pure PLA. The gradient ChNF reached 117% higher energy dissipation when compared to PLA and exhibited a high compressive modulus, which is an interesting behavior, since PLA is typically a very brittle material. However, the best values for toughness and compressive modulus were observed for uniform pore structures, which had pores with the same size as the minimum pores of the gradient structures. The hourglass shape was suggested as an alternative to the cylindrical shape for its ability to increase the contact time of the fluid with the filter during flow-through, and this was the geometry used in the adsorption tests.

**Figure 15:**
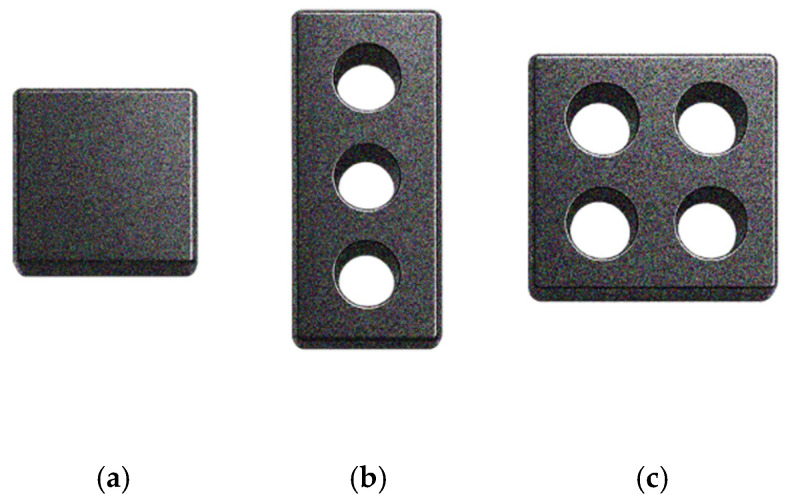
Representative geometries compared by Appuhamillage et al. (adapted from [76]): (**a**) cube, (**b**) shape A, and (**c**) shape B.

Appuhamillage et al. [76] also compared the adsorption capability of a cube, with two perforated block shapes—shape A and shape B of a UV-cured Chitosan/DAP hydrogel (Figure 15)—showing that the solid cube had the lowest adsorption and shape B had the highest, with around 4 times the Pb^2+^ removal of the cube. This was attributed to the higher surface area of shape B, which led to faster and higher adsorption. These findings highlight one of the key advantages of 3DP for the fabrication of such materials, namely the ability to design complex internal channels and porous geometries that enhance the mass transfer and adsorption performance.

From these studies, it is evident that 3DP enables easy fabrication of hierarchical porous structures that enhance the adsorption performance. Furthermore, components designed with larger surface areas that are in contact with water should be prioritized, as this increases the accessibility of adsorption sites and improves the overall adsorption capacity of the material.

### 6.2. Dye Removal

These observations extend to dye removal applications as well. Consequently, several studies have investigated more complex geometries to enhance the contaminant adsorption.

Similarly to what has been reported for heavy metal removal, Ng et al. [117] compared cube and cube-lattice geometries fabricated from PEGDA and PEGDA: HEMA materials to understand the advantage of 3DP. The results showed that the maximum adsorption after 5 h for the solid cubes was below 0.1 mg·g^−1^, whereas the lattice structures reached values slightly below 0.3 mg·g^−1^ for PEGDA and about 0.6 mg·g^−1^ for PEGDA: HEMA. These results highlight the crucial influence of printed geometry on adsorption performance. The lattice structures provide a larger accessible surface area and interconnected pores, which expose more active adsorption sites and reduce mass transfer resistance for dye diffusion within the monolith.

Regarding the macropore size, Li et al. [114] tested NH_2_-MIL-101(Al)-doped PA-12-based films with squares with 1, 2, and 3 mm lateral size for Methylene Blue removal. They concluded that these grids created channels that increased the exposed area of the embedded MOF particles, increasing adsorption. Experimental tests confirmed that the 1 × 1 mm^2^ grid had the fastest adsorption rate, demonstrating that geometry and macropore size or openings can influence the adsorption performance.

Ranjbar et al. [124] developed a monolithic cyclodextrin polymer-g-C_3_N_4_ nanocomposite structure with good mechanical and chemical stability, as well as promising reusability. The study showed that adsorption occurs through host–guest inclusion within the cyclo-dextrin cavities, resulting in different removal efficiencies depending on the size of the dye molecules. Based on this mechanism, the authors suggested that the material may exhibit selectivity toward smaller positively charged dyes, which could provide opportunities for targeted contaminant removal.

**Figure 16:**
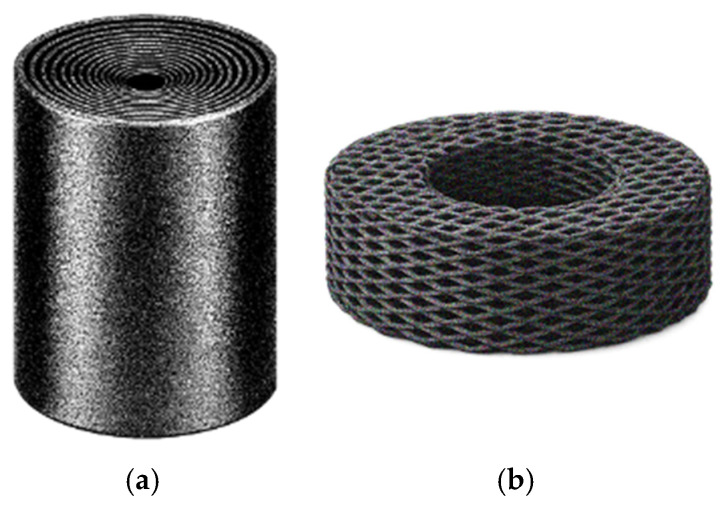
Representative geometries used by (**a**) Delikanli et al. (adapted from [92]) and (**b**) Ortega-Columbrans et al. (adapted from [81]).

Delikanli et al. [92] exploited 3DP to increase the adsorption area and used a spiral filter made from wood-reinforced PLA (Figure 16a), noting that the spiral geometry provides a large surface area within a compact structure. This design also creates a longer flow path for the solution, increasing the residence time of the fluid within the filter and thereby enhancing the contact between the contaminant and the adsorbent material.

Ortega-Columbrans et al. [81] investigated photocatalytic degradation by comparing conventional 2D membranes with 3D-printed scaffolds (Figure 16b) featuring linear and gyroid geometries. The 2D membrane achieved the highest removal efficiency for Methyl Orange (98%); however, since it was not fabricated by 3DP, it was not included in the comparison table (Tables S3 and S4). Among the printed structures, the gyroid scaffolds exhibited a higher removal efficiency (90%) than the linear geometries (60%). This improvement was attributed to the more complex gyroid architecture, which provides a larger contact surface and higher fluid permeability, thereby enhancing photocatalytic degradation.

**Figure 17:**
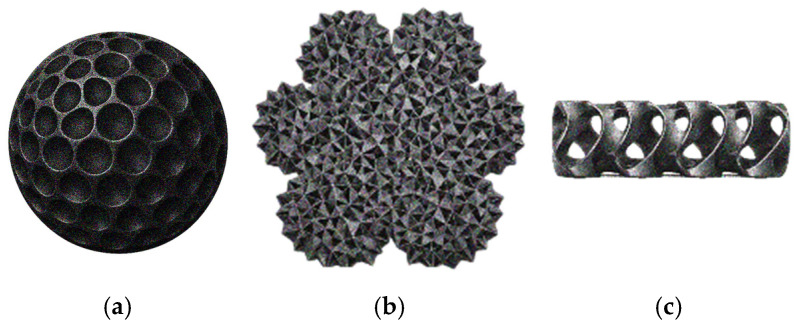
Representative geometries used by (**a**) Shahzadi et al. (adapted from [118]), (**b**) Wang et al. (adapted from [120]) and Li et al. (adapted from [119]), and (**c**) Zhang et al. (adapted from [121]) and Liu et al. (adapted from [122]).

Other studies have also explored the use of more complex geometries to prove that the geometry of the printed adsorbent plays an important role in the dye removal performance. For example, Shahzadi et al. [118] used a trypophobia ball structure: a spherical object containing many regularly distributed holes (Figure 17a). This geometry, similar to the spiral used by Delikanli et al. [92], increases the surface area available for adsorption when compared with a solid structure of a similar size. The porous design also enhances the mass transfer, allowing the dye solution to access both the external and internal surfaces of the material and thereby exposing a larger number of adsorption sites. Additionally, such complex porous architectures can be readily fabricated using SLA, while it would be very difficult to produce through conventional molding or traditional manufacturing techniques.

Also exploiting 3DP abilities to manufacture complex geometries, Wang et al. [120] and Li et al. [119] used fractal geometries (Figure 17b). The main advantage of these architectures lies in their ability to increase the effective surface area available for catalyst deposition, which exposes a larger number of active nanoparticles to the solution and strengthens the interactions between the catalyst and the pollutant molecules. However, the studies also highlight that increasing structural complexity beyond a certain point can become detrimental, as it may limit the light penetration and reduce the activation of catalyst layers located deeper within the structure.

In degradation-based treatment methods, the choice of geometry can contribute not only to improving the reaction efficiency but also to minimizing the risk of secondary contamination. For example, D’Accolti et al. [82] employed a 3D orthogonal lattice structure for the Fenton degradation of Methylene Blue. This architecture exposed a large catalytic surface while keeping the iron oxide catalyst immobilized within the structure, allowing for its easy recovery after treatment and reducing the likelihood of catalyst release into the treated water.

Zhang et al. [121] used an ABS/TPU/CaSiO_3_ composite filament with the growth of ZnO nanospheres during hydrothermal synthesis. Later, Liu et al. [122] used the same ABS/TPU paired with *chlorella*-assisted Fe_2_O_3_. For both studies, the chosen structure is a complex skeleton part (Figure 17c), produced by FDM, which not only provides a high surface area and enables effective contact, but also a local dye-enrichment zone on the surface, enhancing photocatalytic degradation. Additionally, as noted by D’Accolti et al. [82], this geometry also allows for the easy recovery and reuse of the catalyst material.

Wang et al. [113] investigated ABS parts coated with the MOF Cu-BTC, where Cu-BTC served as the primary adsorbent. In such cases, the adsorbent was applied only as a surface coating, making the presence of macropores within the printed structure or having a complex geometry particularly important. A porous architecture allows the coating to penetrate the internal structure, increasing the available adsorption surface. Without such porosity, the adsorbent would be limited to a thin external layer, significantly restricting the effective active surface area. 3D orthogonal lattice structures were printed to act as structured supports that allow water to flow easily around the MOF-coated surfaces. This structured design enabled fast dye removal (within about 10 min).

**Figure 18:**
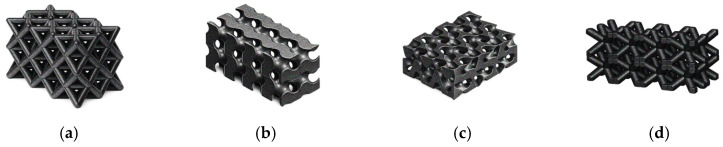
Representative geometries used by Yusoff et al. (adapted from [108]): (**a**) Kagome, (**b**) Gyroid, (**c**) Fisher Koch, and (**d**) BCC.

Yusoff et al. [108] studied the effect of geometry on Methyl Orange removal, using a Chitosan/PEDGA material developed in a previous study [107]. For that, four different geometries were studied: Kagome, Gyroid, Fisher Koch, and body-centered cubic (BCC) (Figure 18), with increasing specific surface area in this order, with Fisher Koch and BCC having a similar value. In terms of permeability, the order was the same, but the value for BCC was almost two times that of Fisher Koch. Regarding the removal capacity and adsorption capacity, the order was also the same, establishing BCC as the best geometry, as concluded in this work. However, this is the geometry with the worst mechanical properties, with the best one being Fischer (1.7 times the yield strength and 3.5 times the Young’s modulus of the BCC); therefore, in a flow-through application, where the mechanical properties of the structure are a restriction for its durability, this can be an interesting alternative.

Cirillo et al. [87] used three different electrode geometries to remove MB from water using a PLA/Carbon Black filament—a single spiral, a double spiral and a triple spiral—concluding that the single spiral provided good fluid circulation around the electrode; however, due to its smaller surface area, it had a smaller catalytic surface area, removing only 89% of the dye. Two spirals improved the catalytic potential due to the increased surface area, making the removal potential 97%. However, increasing to three spirals made the flow too complex, forming dead zones and accumulation of gas bubbles, and reducing the removal potential to 69%.

## 7. Current Limitations and Future Perspectives

The analysis of this problem can be divided into two main areas: material design and geometric optimization, and the general effects.

First, regarding material design, there is a clear effort to incorporate more sustainable materials, which represents a promising direction for the development of environmentally responsible treatment systems. However, despite this progress, further improvements in the adsorption performance remain necessary, particularly to reduce equilibrium and contact times, enabling effective contaminant removal under realistic flow conditions, since there are many solutions that already have interesting removal capacities, but very long contact times are still required. Faster adsorption kinetics would allow for the design of gradient or staged structures, in which each section of the component removes a fraction of the contaminant, progressively reducing its concentration along the flow path until safe levels are reached at the outlet.

Still, achieving faster kinetics must be balanced with material stability. The sustainable polymers alternatives explored often exhibit moderate stability under the harsh conditions typically encountered in water treatment environments, particularly during exposure to acidic or alkaline media. Additionally, the proposed regeneration processes frequently rely on the use of strong acids, which can further compromise the material integrity over time. Even when materials demonstrate improved stability, concerns remain regarding the use of functionalization agents, as well as the potential leached compounds, residual monomers, and degradation products.

Many of the most effective solutions reported rely on the incorporation of nanomaterials to enhance the adsorption capacity or to promote degradation processes, with GO and MOF being the most common. These materials, although very interesting, may be dangerous, since they may be released into treated water because of mechanical abrasion induced by flow conditions, chemical degradation, or incomplete immobilization within the polymer. Such a release can contribute to secondary contamination and raise safety concerns for both aquatic ecosystems and human health. Therefore, more comprehensive safety assessments, including chemical stability testing and life cycle assessment, are required to better understand the potential environmental impacts and to support the safe and sustainable implementation of these materials in practical water treatment applications.

Finally, a direct comparison between studies remains difficult, since experimental conditions vary widely, including geometry, contaminant concentration, adsorbent mass, solution volume, pH, and temperature. These variations make it difficult to reliably assess performance and to identify which solutions are truly more effective under comparable conditions. The lack of consistency in experimental design therefore limits the ability to draw more meaningful conclusions across studies. The development and adoption of standardized testing protocols would improve the reproducibility and enable more robust comparison between materials and system configurations.

Regarding geometry, most current studies primarily explore different material formulations and removal mechanisms, while the geometries used in the printed components are often simplified, such as 3D orthogonal lattices, which are mainly intended to support material evaluation, rather than to achieve optimized removal or structural performance. Therefore, the role of geometry should also be addressed. Systematic research should be conducted in which geometric parameters, such as the overall architecture, pore size, infill patterns, and internal channel structure, are varied while maintaining the same material composition, as the current design choices are frequently based on empirical assumptions, and direct comparisons between configurations remain limited. This approach would allow for the direct evaluation of how structural design influences the mass transfer, contact area, and overall removal efficiency. Additionally, this would be essential to better understand the mechanical stability and long-term durability of these systems. More comprehensive mechanical characterization should therefore be conducted, including testing not only under dry conditions but also after prolonged water exposure, aging, and cyclic loading, as most applications involve continuous-flow operation. These systems could then be evaluated using computational fluid dynamics (CFD) simulations to assess the fluid flow, mass transfer, and operational performance and durability under realistic conditions.

When considering the whole system, several gaps in the experimental design become evident, as most studies rely primarily on batch experiments, while continuous-flow operation, which potentially represents the most common configuration in industrial water treatment systems, is less frequently investigated, resulting in a limited understanding of the mechanical stability, durability, and adsorption performance under realistic operating conditions, limiting the ability to predict the system performance at larger scales.

From the technologies used, FFF represents an interesting pathway, as it is widely accessible and capable of producing components with sufficient geometrical stability. Furthermore, what is typically considered its main limitation, the formation of printing defects, can be an advantage for this application, enabling the creation of hierarchical porosity. Technologies such as DIW and SLA are also analyzed, but their use can be challenging in terms of scalability for industrial water treatment applications, due to their relatively slow production rates, material formulation constraints, and the need for post-processing steps. For these technologies, in general, the feasibility of large-scale manufacturing and long-term operational stability remain insufficiently evaluated.

Finally, building on the developments in material and geometry, future work should also focus on the development of integrated components designed for real-world operation. For example, devices could incorporate complex architectures or different geometrical features along the flow path, allowing distinct regions of the component to perform different functions, such as adsorption, catalytic degradation, or filtration. Additionally, 3DP technologies enable the possibility of fabricating multi-material structures at the macroscopic scale. For instance, FFF systems equipped with multiple nozzles can print components composed of two or more materials within a single structure. This capability could be used to target different classes of contaminants simultaneously, with each material being optimized for the removal of specific pollutants.

## 8. Conclusions

In summary, 3DP is a promising strategy for producing components for water treatment applications aimed at contaminant removal, as it allows for precise control over parameters such as porosity and geometry, enabling the fabrication of tailored components. It is also highly flexible, as it is compatible with a wide range of materials.

The polymeric materials used are typically functionalized or combined with additives, with GO and MOFs being the most common. Systems in which both the polymer matrix and the additives actively contribute to contaminant removal generally exhibit the best performance. Additionally, there is a clear effort to maintain the sustainability of the final structure, using biopolymers or biodegradable synthetic polymers, which can serve both as active materials and as structural supports.

3DP enables the fabrication of complex geometries that maximize the surface area in compact components. Furthermore, it allows for the incorporation of macroporosity, which facilitates fluid flow through the structure and increases contact between the contaminant and the material. At the same time, the polymeric materials should possess mesopores and micropores to increase the availability of adsorption sites: a benefit that can be further enhanced by the swelling capacity of certain polymers. This hierarchical pore structure has been consistently identified as being critical for achieving high removal efficiency.

The primary removal mechanism for heavy metals is adsorption, which is also highly effective for cationic dyes, as most polymer surfaces are negatively charged. However, for anionic dyes, surface modification or the incorporation of additional removal mechanisms, such as degradation processes, is often required. Although effective, these processes are less suitable for practical applications, particularly in continuous-flow systems, as they typically require external activation or the addition of chemical agents that may lead to secondary pollution.

Although several systems have demonstrated promising removal capacities and regeneration performance, most still require long contact times and have only been tested using simplified or reference geometries. Further work is needed to develop practical devices that are suitable for continuous-flow operation.

## Data Availability

Not applicable.

## Supplementary Materials

The following supporting information can be downloaded at: https://www.mdpi.com/article/10.3390/polym18091029/s1, Table S1: PRISMA Checklist [125]; Figure S1: PRISMA flow diagram of the literature screening and selection process; Table S2: Search query divided into conceptual blocks; Table S3: Documents on the removal of heavy metals; Table S4: Documents on the removal of dyes.

## Abbreviations

The following abbreviations are used in this manuscript:

3DP	Three-dimensional printing
ABS	Poly(acrylonitrile–butadiene–styrene)
AM	Additive manufacturing
BCC	Body-centered cubic
BSA	Bovine serum albumin
CA	Calcium Alginate
CFD	Computational fluid dynamics
ChNF	Chitin nanofibers
CNC	Cellulose nanocrystals
COF	Covalent–organic frameworks
DAP	Diacrylated Pluronic F-127
DIW	Direct ink writing
DLP	Digital light processing
DMSA	2,3-dimercaptosuccinic acid
FFF	Fused filament fabrication
GO	Graphene oxide
HAp	Hydroxyapatite
HEMA	2-hydroxylethyl methacrylate
LCD	Liquid crystal display
MCC	Microcrystalline cellulose
MIP	Molecularly imprinted polymers
MOF	Metal–organic frameworks
PA	Polyamide
PAA	Poly(acrylic acid)
PBAT	Poly(butylene adipate-co-terephthalate)
PBS	Poly(butylene succinate)
PCL	Polycaprolactone
PDA	Polydopamine
PEGDA	Poly(ethylene glycol diacrylate)
PEI	Polyethyleneimine
PEO	Poly(ethylene oxide)
PLA	Poly(lactic acid)
POM	Polyoxometalate
PP	Polypropylene
PS	Polystyrene
PVA	Poly(vinyl alcohol)
PVDF	Poly(vinylidene fluoride)
PVPA	Poly(vinyl phosphonic acid)
SA	Sodium Alginate
SLA	Stereolithography
SLS	Selective laser sintering
TEA	Triethylamine
TOCNF	TEMPO-oxidized cellulose nanofiber
TPU	Polyurethane
UV	Ultraviolet
