# Supplementary material for: The Use of 3D-Printed Polymer Components for the Removal of Heavy Metals and Dyes from Water: A Systematic Literature Review
Source: Polymers (Basel). 2026 Apr 24;18(9):1029. doi: 10.3390/polym18091029 (PMC13165334; doi:10.3390/polym18091029)
Supplement: Supplementary file 1 [file polymers-18-01029-s001.zip › Table S4_dye_table.pdf]

Reference	3DP	Polymer	Additives	Contaminant	Method	Removal efficiency / %	Time	Regeneration (retained capacity)	Kinetics	Isotherm	Quality score
Ranjbar et al. [124]	DLP	CDP-g-C3N4		MB+, RhB, MO, CR	Adsorp.	91	90 min	5 cycles (78%)	PSO	Langmuir	1.8
Pereira et al. [112]	FFF	PLA		MR, IC, RB5	Ultrafiltration, Adsorp.	98	-	2 cycles (similar)	-	-	1.6
Cirillo et al. [86]	FFF	PLA	Carbon Black, Silver	MO	Electrochem. Degrad.	100	60 min	5 cycles	-	-	1.7
Cirillo et al. [87]	FFF	PLA	Carbon Black, Cu	MB+	Electrochem. Degrad.	97	60 min	10 cycles	-	-	1.7
Dhillon et al. [79]	FFF	PLA	Bismuth ferrite	CR, MB	Photochem. Degrad.	99	90 min	5 cycles (97%)	-	-	1.6
Ranjbar et al. [80]	DIW*	Cellulose (Phosphorylated)	MoS2	MB+	Adsorp., Photochem. Degrad.	99	120 min	5 cycles (91%)	PSO	Langmuir	1.7
Delikanli et al. [92]	FFF	PLA	Wood	CV	Adsorp., Filt.	97.50	-	3 cycles (95.8%), 7 cycles (51.4%)	-	-	1.6
Zheng et al. [89]	FFF	PP, ABS		MB+, CV, AO7	Adsorp., Oxid. Degrad.	78	50	-	PFO	Langmuir	1.8
Ortega-Columbrans et al. [81]	FFF	PLA	TiO2	MO	Adsorp., Photochem. Degrad.	90	- (24 h)	-	-	-	1.4
Alves et al. [109]	DIW*	SA, Carboxymethylcellulose, Gelatin	MOF (MIL-88A), MOF(Fe based), MoS2	MB+	Adsorp., Photochem. Degrad.	95.86	24 h	5 cycles (58%)	PSO		1.6
Ranjbar et al. [49]	DIW*	Cellulose (Phosphorylated)		MB+	Adsorp.	99	-	7 cycles (similar)	PSO	Langmuir	1.5
Liu et al. [122]	FFF	ABS, TPU	Chlorella powder, Iron Oxide	MO	Adsorp., Photochem. Degrad.	91	4 h	6 cycles (99%)	-	-	1.6

Continued on next page

Reference	3DP	Polymer	Additives	Contaminant	Method	Removal efficiency / %	Time	Regeneration (retained capacity)	Kinetics	Isotherm	Quality score
Ng et al. [117]	LCD	Crosslinked acrylic network (PEGDA:HEMA)		RhB	Adsorp.	94.86	5 h	5 cycles (50%)	PSO	Redlich-Peterson	2
Bößl et al. [88]	DIW*	PVDF		RhB	Sonochem. degradation, Adsorp.	>95	40 min	-	-	-	1.1
Mohd Yusoff et al. [108]	DLP	PEGDA, Chitosan		MO	Adsorp., Filt.	90.40	2	-	-	-	1.4
Abdelhamid et al. [50]	DIW*	Cellulose (TOCNF)	MOF (ZIF-8), Hmim, ZnO	MB-, RhB	Adsorp., Catalys.	100	10 min	-	-	-	1.3
Fijol et al. [51]	FFF	Cellulose (TOCNF), PLA	MOF (SU-101)	MB+	Adsorp., Filt.	57	- (24 h)	-	-	-	1.6
Shahzadi et al. [118]	SLA	Thiol–yne polymer (BA/PETMP)		MG	Adsorp.	100	10 min	6 cycles (100%)	-	Langmuir	1.9
D'Accolti et al. [82]	FFF	PLA, PVA	Fe oxides	MB+	Fenton Degrad.	100	30 min	3 cycles	-	-	1.6
Park et al. [111]	FFF	PLA	GO	MB+	Adsorp., Filt.	75	6 h	-	PSO	Langmuir	1.8
Mohd Yusoff et al. [107]	DLP	PEGDA, Chitosan		MO	Adsorp., Filt.	90.40	2 h	4 cycles (78%)	PSO	Langmuir	2
Wang et al. [120]	LCD	PVPA	Zn-doped CdS	RhB	Adsorp., Photochem. Degrad.		-	4 cycles (96.3%)	-	-	1.6
Shojaeiarani et al. [106]	DIW*	PEO, Cellulose		MB+, MG	Adsorp.	95.23	10 h		PSO	-	1.4
Zheng et al. [116]	FFF	PVA, PLA, PBS	Camellia Seed Powder	MB+	Adsorp.	99.44	22h	7 cycles (99%)	PSO	Langmuir	2
Masud et al. [54]	DIW	PDA, BSA	Graphene	MB+, EB	Adsorp., Filtration	99	- (total 96 h)	-	-	Langmuir	1.7
Zhang et al. [121]	FFF	ABS, TPU	CaSiO3, ZnO	RhB	Adsorp., Photochem. Degrad.	97.94	- (8 h)	5 cycles (>90%)	PFO	-	1.7

Continued on next page

Reference	3DP	Polymer	Additives	Contaminant	Method	Removal efficiency / %	Time	Regeneration (retained capacity)	Kinetics	Isotherm	Quality score
Li et al. [119]	FFF	PLA, PAA	ZnO, TiO2, MOF (Fe-BTC)	RhB	Adsorp., Photo-chem. Degrad.	94.30	- (6 h)	3 cycles (89%)	PFO	-	1.7
Xia et al. [115]	FFF	PLA, PBAT	Chlorella pyrenoidosa	MB+	Adsorp.	92.66	800 min	6 cycles (78%)	PSO	Langmuir	2
Li et al. [114]	SLS	PA-12	MOF (ZIF-67), MOF(NH2-MIL-101(Al)), MOF-801, MOF(HKUST-1), MOF(ZIF-8)	MB+	Adsorp.	95%	- (24 h)	5 cycles (85%)	-	Langmuir	1.9
Shi et al. [110]	FFF*	PLA	MOF (Cu-based)	MG	Adsorp.	93%	10 min	3 cycles (90%), 5 cycles (70%)	-	-	1.6
Wang et al. [113]	FFF*	ABS	MOF (Cu-BTC)	MB+	Adsorp.	98.3	10 min	5 cycles (62%)	-	-	1.8

Methylene Blue (MB+), Rhodamine B (RhB), Methyl Orange (MO), Congo Red (CR), Methyl Red (MR), Indigo Carmine (IC), Reactive Black 5 (RB5), Crystal Violet (CV), Acid Orange 7 (AO7), Methyl Blue (MB-), Malachite Green (MG), Evans Blue (EB) * Not directly mentioned by the author
