# Supplementary material for: The Use of 3D-Printed Polymer Components for the Removal of Heavy Metals and Dyes from Water: A Systematic Literature Review
Source: Polymers (Basel). 2026 Apr 24;18(9):1029. doi: 10.3390/polym18091029 (PMC13165334; doi:10.3390/polym18091029)
Supplement: Supplementary file 1 [file polymers-18-01029-s001.zip › Table S2_query_table.pdf]

Concept	Search query block
Indicate contaminant removal	remov* OR treat* OR degrad* OR separ* OR oxid* OR adsorb* OR adsorp* OR biosorption OR uptake OR removal OR sequestration OR capture
Indicate target contaminants	metal* OR heavy metal* OR metallic ion* OR metalic ion* OR lead OR Pb OR iron OR Fe OR cadmium OR Cd OR copper OR Cu OR nickel OR Ni OR zinc OR Zn OR cobalt OR chromium OR Cr OR mercury OR Hg OR arsenic OR manganese OR Mn OR vanadium Or V OR dye* OR oil*
Indicate material / structure	polymer* OR biopolymer* OR PLA OR “poly(lactic acid)” OR ABS OR “poly(acrylonitrile-butadiene-styrene)” OR PETG OR “poly(ethylene terephthalate glycol)” OR PCL OR “polycaprolactone” OR PP OR “polypropylene” OR PEEK OR “poly(ether ether ketone)” OR PSU OR “polysulfone” OR PES OR “polyethersulfone” OR PVDF OR “pol(vinylidene fluoride)” OR TPU OR “thermoplastic polyurethane” OR PA OR “polyamide” OR nylon OR chitosan OR alginate OR “sodium alginate” OR cellulose OR “carboxymethyl cellulose” OR gelatin OR starch OR “photopolymer resin*” OR PVA OR “poly(vinyl alcohol)” OR PEG OR “poly(ethylene glycol)” OR PEGDA OR “poly(ethylene glycol diacrylate)” OR PAA OR “poly(acrylic acid)” OR PAM OR “polyacrylamide” OR HEMA OR “2-hydroxyethyl methacrylate” OR hydrogel*
Indicate manufacturing method	“3D print*” OR “3-dimensional print*” OR 3DP OR “three-dimensional print*” OR “additive manufactur*” OR “rapid prototyping” OR “material extrusion” OR “fused deposition modeling” OR FDM OR “fused filament fabrication” OR FFF OR stereolithograph* OR SLA OR “selective laser sintering” OR SLS OR DLP OR “digital light processing” OR “vat photopolymerization” OR “direct ink writing” OR DIW OR inkjet printing
Indicate water medium	water OR wastewater OR aqueous

All the concepts were searched in Title or Abstract, which corresponds to the fields TI=(...) OR AB=(...) in Web of Science and TITLE(...) OR ABS(...) in Scopus. This approach helped minimize differences in the search strategy between the two databases. The results were limited to research articles written in English. For the metal contaminants, both the general terms and specific heavy metals were used in the search, and, for all of them, both element name and its chemical symbol were used, except for arsenic and cobalt. Including “As” led to around 1,000 additional results, which were false positives due to the conjunction “as” in the title or abstract. Similarly, including “Co” would lead to false positives due to the prefix “co”. Iron was also considered in the search, although, unlike the other mentioned metals, it is not generally considered toxic at typical concentrations, however, since it is one of the most abundant metals in water systems, studying methods for iron removal may provide useful findings for strategies that can be applied to other metal ions. For dye contaminants, all results containing the term “dye” were considered, as this term is typically present even when a specific dye compound is mentioned.
