# Supplementary material for: The Use of 3D-Printed Polymer Components for the Removal of Heavy Metals and Dyes from Water: A Systematic Literature Review
Source: Polymers (Basel). 2026 Apr 24;18(9):1029. doi: 10.3390/polym18091029 (PMC13165334; doi:10.3390/polym18091029)
Supplement: Supplementary file 1 [file polymers-18-01029-s001.zip › Table S3_heavymetal_table.pdf]

Reference	3DP	Polymer	Additives	Contaminant	Method	Capacity / mg g^{-1}	Time	Regeneration (retained capacity)	Kinetics	Isotherm	Quality score
Ibebunjo et al. [42]	SLS	Cellulose (MCC), PA		Pb	Adsorp.	19.82 (filter), 66.1 (MCC)	4 h	5 cycles (85%)	PSO	Langmuir	2
Zhang et al. [58]	DIW	Chitosan	GO, SiO2	Cu	Adsorp.	300	180 min	6 cycles (70%)	PSO	Langmuir	1.8
Joseph et al. [99]	SLS	Chitosan, PS	MOF (CS-MIL-100)	As, As	Adsorp., Filt.	4.5 (filter), 30.2 (filler)	- (2 h)	-	-	Freundlich	1.7
Zhang et al. [45]	DIW	PVA, SA	GO	Cu	Adsorp.	206	- (400 min)	5 cycles (72%)	PSO	Langmuir	1.8
Zhang et al. [68]	DIW	SA, Cellulose	GO	Cu	Adsorp.	250	90 min	5 cycles (80%)	PSO	Langmuir	1.8
Swathe Sriee et al. [96]	DIW*	SA, Hyaluronic Acid	Fungi (Aspergillus flavus)	Cu, Cd, Ni, Co, Fe	Adsorp.	- (97.59%)	12 h	-	-	-	1.1
Asghartabar Kashi et al. [62]	DIW*	PVA, PAA		Pb	Adsorp.	896	2 min	-	PSO	Freundlich	1.3
Wu et al. [63]	SLA	Chitosan	ZIF-67	Pb	Adsorp., Filt.	5.47	- (240 min)	-	PSO	Langmuir	1.6
Kanaan et al. [103]	FFF	PVA, PU		Cu, Fe, Zn	Adsorp.	- (>80%)	68 h	-	-	-	1.4
Zhang et al. [67]	DIW	Chitosan	GO	Cu	Adsorp.	269	210 min	6 cycles (71%)	PSO	Langmuir	1.8
Burratti et al. [105]	DLP*	PEGDA	Silver	Hg	Adsorp., Filt.	0.61	8 h	-	MO	Langmuir	1.3
Rezanavaz et al. [123]	DPL	Acrylic-based MIP		Cu	Adsorp.	-	-	-	-	Langmuir	1.3
Park et al. [55]	FFF	PLA	GO	Cd	Adsorp., Filt.	1.26	60 min	5 cycles (68%)	PSO	Langmuir	2
Abdelhamid et al. [46]	DIW	Cellulose (TOCNF), SA	COF-1, COF-2	Al, Co, Cr, Cd, Cu, Fe, Ni, Zn	Adsorp.	410.8	180 min	3 cycles (similar)	-	-	1.5
Wang et al. [95]	DIW	Calcium Alginate	GO	Pb	Adsorp., Filt.	490.2	4h	8 cycles (81%)	PSO	Freundlich	2
Wang et al. [101]	FFF	PLA, Chitosan	Hydroxyapatite	Cu	Adsorp., Filt.	119	300 min	5 cycles (98%)	PSO	Langmuir	2
Wu et al. [43]	DIW*	SA, Cellulose (CNC)		Cu	Adsorp.	97.22	-	5 cycles (88%)	PSO	Langmuir	1.5
Abdelhamid et al. [50]	DIW*	Cellulose (TOCNF)	MOF (ZIF-8), Hmim, ZnO	Co, Cu	Adsorp.	328	- (12 h)	-	-	-	1.3
Fijol et al. [51]	FFF	Cellulose (TOCNF), PLA	MOF (SU-101)	Cd, As, Pb, Mn, Zn	Adsorp., Filt.	- (70-80%)	- (24 h)	3 cycles (similar/higher)	-	-	1.6
Zhang et al. [47]	DIW	SA	GO	Cu	Adsorp.	179.32	- (400 min)	5 cycles (74.6%)	PSO	Langmuir	1.8

Continued on next page

Reference	3DP	Polymer	Additives	Contaminant	Method	Capacity / mg g^{-1}	Time	Regeneration (retained capacity)	Kinetics	Isotherm	Quality score
Fijol et al. [56]	FFF	PLA, Cellulose (TOCNF), Chitin		Cu	Adsorp., Filt.	12.6 (filter), 234 (filler)	- (8 h)	3 cycles (20%)	-	-	1.8
Lan et al. [102]	FFF	PVA, PU	Chlorella pyrenoidosa	Pb	Adsorp., Filt.	- (75.61)	48 h	7 cycles (similar/higher)	-	-	1.6
Finny et al. [57]	DIW*	SA, Gelatin, PEI		Cu, Ni, Cd, Co, Pb	Adsorp.	633.3 (dehydrated), 43.52 (hydrated)	18 h	-	-	Freundlich	1.4
Miao et al. [64]	DIW	Gelatin, SA	Montmorillonite nanosheets	Pb	Adsorp., Filt.	134	240	5 cycles (49%)	PSO	Freundlich	2
Thakare et al. [93]	DIW*	Alginate, Methylcellulose	Algae	Cu	Adsorp., Filt.	-	2 h	-	-	-	1.1
Fijol et al. [100]	FFF	PLA	Hydroxyapatite	Cd, Pb	Adsorp., Filt.	360.5 (filler), 54 (calc. filter)	- (12 h)	-	-	-	1.6
Zhang et al. [54]	DIW	PDA, BSA	Graphene	Cr, Pb	Adsorp.	45.05	- (96 h)	-	-	Langmuir	1.7
Liakos et al. [44]	FFF	PCL, SA		Cu	Adsorp.	93.3	30 days	-	-	-	1.6
Shahbazi et al. [75]	DIW*	SA, PAA	Nanoclay	Pb	Adsorp.	532	15 min	-	PSO	Freundlich	1.3
Appuhamillage et al. [76]	DIW*	Chitosan, DP f-127		Pb, Cu, Cd, Hg	Adsorp., Filt.	0.6	30 min	5 cycles (98%)	-	-	1.5
Ji et al. [104]	FFF*	ABS	POM anions	Co, Ni, Cu	Adsorp., Filt.	-	12 h	-	-	-	1.4

* Not directly mentioned by the author
