# Supplementary figures and images for: The Use of 3D-Printed Polymer Components for the Removal of Heavy Metals and Dyes from Water: A Systematic Literature Review
Source: Polymers (Basel). 2026 Apr 24;18(9):1029. doi: 10.3390/polym18091029 (PMC13165334; doi:10.3390/polym18091029)

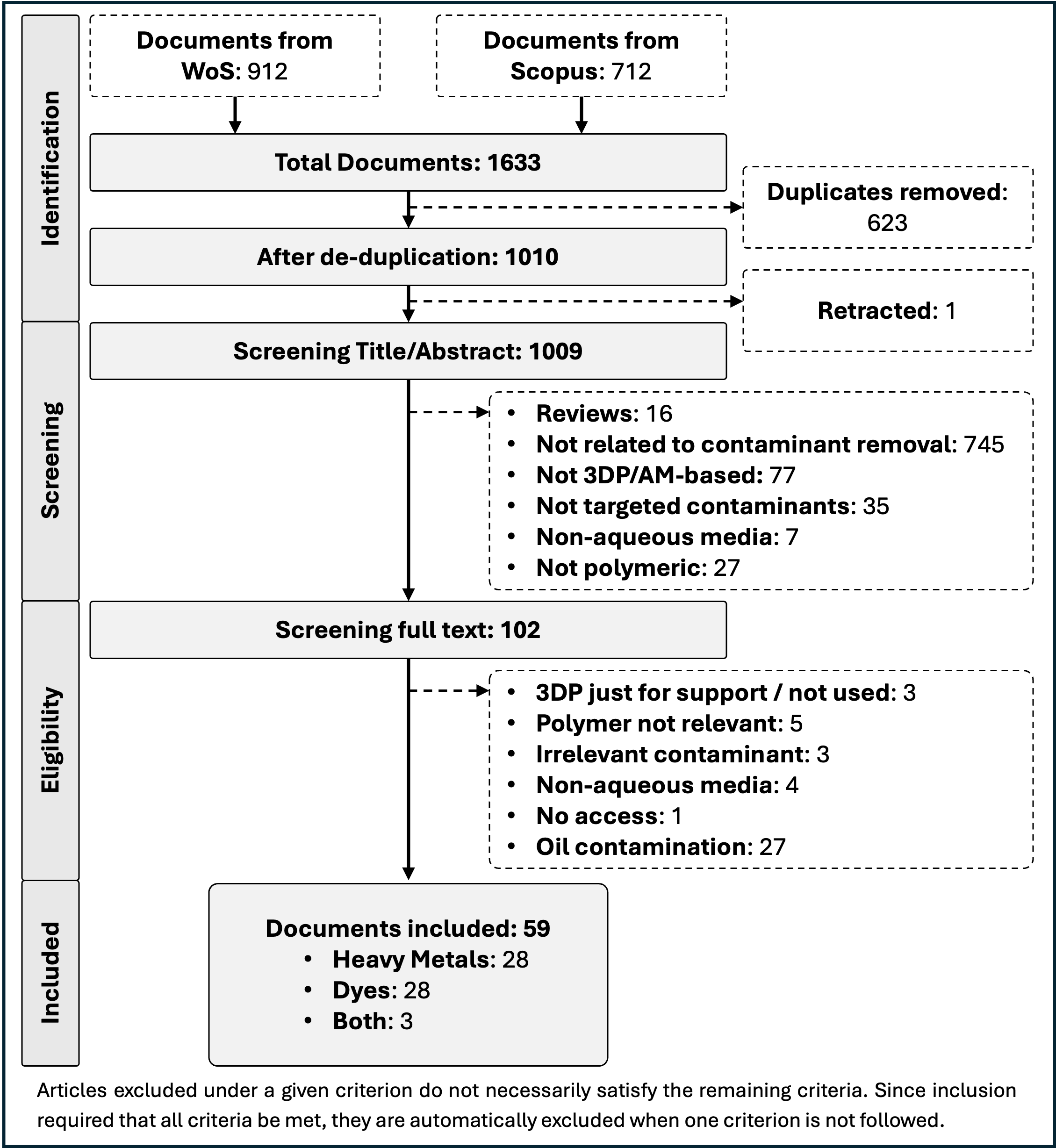

Supplement: Supplementary file 1 [file polymers-18-01029-s001.zip › Figure S1_PRISMA.png]
